# Phase heterogeneity in carbonate production by marine fish influences their roles in sediment generation and the inorganic carbon cycle

**DOI:** 10.1038/s41598-017-00787-4

**Published:** 2017-04-10

**Authors:** Michael A. Salter, Alastair R. Harborne, Chris T. Perry, Rod W. Wilson

**Affiliations:** 1 Geography, College of Life and Environmental Sciences, University of Exeter, Amory Building, Rennes Drive, Exeter EX4 4RJ UK; 2 Department of Biological Sciences, Florida International University, North Miami, Florida 33181 USA; 3 Marine Spatial Ecology Laboratory and Australian Research Council Centre of Excellence for Coral Reef Studies, School of Biological Sciences, Goddard Building, The University of Queensland, Brisbane, QLD 4072 Australia; 4 Biosciences, College of Life and Environmental Sciences, University of Exeter, Geoffrey Pope Building, Stocker Road, Exeter, EX4 4QD UK

## Abstract

Marine teleost fish are important carbonate producers in neritic and oceanic settings. However, the fates of the diverse carbonate phases (*i.e*., mineral and amorphous forms of CaCO_3_) they produce, and their roles in sediment production and marine inorganic carbon cycling, remain poorly understood. Here we quantify the carbonate phases produced by 22 Bahamian fish species and integrate these data with regional fish biomass data from The Bahamas to generate a novel platform-scale production model that resolves these phases. Overall carbonate phase proportions, ordered by decreasing phase stability, are: ~20% calcite, ~6% aragonite, ~60% high-Mg calcite, and ~14% amorphous carbonate. We predict that these phases undergo differing fates, with at least ~14% (amorphous carbonate) likely dissolving rapidly. Results further indicate that fisheries exploitation in The Bahamas has potentially reduced fish carbonate production by up to 58% in certain habitats, whilst also driving a deviation from natural phase proportions. These findings have evident implications for understanding sedimentary processes in shallow warm-water carbonate provinces. We further speculate that marked phase heterogeneity may be a hitherto unrecognised feature of fish carbonates across a wide range of neritic and oceanic settings, with potentially major implications for understanding their role in global marine inorganic carbon cycling.

## Introduction

Marine bony fish have recently been identified as a globally important source of marine calcium carbonate^1^. Thus, alongside coccolithophores, foraminifera, and pteropods in oceanic settings, and benthic organisms (such as corals, coralline algae, and echinoderms) and microbial calcification in neritic environments^2–7^, they are now recognised as playing a potentially key role in the marine inorganic carbon cycle^1, 8, 9^. Whilst the bicarbonate transport mechanisms which drive the formation of carbonates in the piscine intestine are now reasonably well understood^10, 11^, and progress has been made in understanding their potential sedimentary significance in shallow warm-water settings^12–14^, the cycling of these carbonates through the marine inorganic carbon system remains poorly understood. This, however, is a significant issue given that: (i) the inorganic carbon cycle, together with the organic carbon cycle, strongly regulates oceanic carbon distribution; and (ii) the contribution to this inorganic carbon cycle by fish is evidently significant: highly conservative modelled estimates suggest they produce 3–15% of global marine carbonates, with less conservative criteria suggesting up to 45%^1^. It is thus important that the role of marine teleosts in the carbon cycle is properly understood and appropriately integrated within ocean carbon models.

Conventional understanding of the inorganic carbon cycle holds that carbonate precipitation takes place primarily in the euphotic zone of oceanic settings, where coccolithophores, foraminifera (calcite) and pteropods (aragonite) are thought to be the major sources, with smaller contributions of aragonite and high-Mg calcite within neritic environments^5, 6^. The net effect of this precipitation can be described by the following reversible reaction:

1 $$C{a}^{2+}+2HC{O}_{3}^{-}\leftrightarrow CaC{O}_{3}+C{O}_{2}+{H}_{2}O$$

Calcification thereby contributes CO_2_ (some of which may be taken up by net organic production) to surface waters and lowers alkalinity, with ~0.6 mol of CO_2_ effectively being generated for each one mole of CaCO_3_ precipitated^15^, whilst carbonate dissolution reverses this process. Calcite and aragonite are stable under most surface water conditions, with dissolution theoretically proceeding only below their saturation horizons at depths greater than ~0.5–4.5 km (depending on carbonate phase and geographic region)^16^. Thus, the fate of most carbonates should either be accumulation as sediment if deposited above their respective saturation horizons, or dissolution in deeper ocean settings; surface water alkalinity remaining depleted in either case. In this context, initial assumptions that fish carbonates are composed entirely of high Mg-calcite (HMC; 5–25 mol% MgCO_3_) and very high Mg-calcite (VHMC; >25 mol% MgCO_3_) formed the basis of a hypothesis wherein they strongly influence alkalinity–depth profiles in the ocean^1^. This is because the solubilities of these metastable carbonate phases exceed those of aragonite (except where MgCO_3_ content is less than ~12 mol%) and calcite^17, 18^, implying that their corresponding saturation horizons are located at shallower depths. Accordingly, this hypothesis invokes fish carbonates–as the only known high-solubility carbonate phases significant to marine carbonate production in oceanic settings–as a unique source of alkalinity in the upper water column that can at least partially explain a widespread positive alkalinity anomaly at depths of ~1000 m^1, 8^.

In shallow marine settings HMC often forms a significant component of accumulated carbonate sediments^19–21^, with fish now seen as a potentially important source of fine-grained carbonate mud (<63 µm)^11–13^. However, several recent studies have demonstrated that fish carbonates produced in shallow water habitats of sub-tropical regions actually occur in a remarkably diverse range of phases. In addition to HMC and VHMC, these include low-Mg calcite (LMC; 0–5 mol% MgCO_3_), aragonite, and amorphous calcium and magnesium-rich carbonate (ACMC)^12, 13, 22^. If established solubility relationships for these phases apply (*i.e*., in order of decreasing solubility: ACMC > VHMC > HMC > aragonite > LMC; solubility in calcites being positively correlated with MgCO_3_ content^19, 23^), fish-derived carbonate solubilities thus span two orders of magnitude, suggesting they will have markedly different post-excretion fates. This implies that their roles in carbonate sediment cycling and inorganic carbon cycling in these neritic environments, and potentially also in oceanic settings if phase heterogeneity applies universally, will strongly depend on what phases are being produced and in what ratios. However, a lack of quantitative data regarding the relative abundances of carbonate phases produced by different fish species and communities–both in neritic and oceanic settings–severely limits our capacity to understand the nature of these roles. Developing such an understanding may be important not only for modelling the role of fish in past, present and future sedimentary scenarios, but also for predicting the oceanic response to rising levels of atmospheric CO_2_, and potentially for informing decisions on how fish stocks should be managed as a carbon-regulating service^9, 24^ in the face the challenges such as climate change mitigation.

Here we utilise attenuated total reflectance Fourier transform infrared (ATR-FTIR) spectroscopy to provide the first quantification of precipitate phases produced by a range of Caribbean fish species. This methodology provides additional capability over previously used X-ray diffraction (XRD) techniques^1, 12, 13^ to enable the presence of both amorphous and crystalline carbonate phases to be determined. Results are combined with morphological and compositional data to facilitate an assessment of the abundances of different phases produced by each species. We then combine these data with production rate data and regional fish census data from a range of habitats in shallow-water areas of the extensive banks of carbonate sediment that form The Bahamas (*i.e*., the Bahamian platform) to model fish carbonate production with respect to different carbonate phases. Because precipitation products vary among fish species^12, 13^, this model not only estimates phase abundances at an archipelago scale; it also models variations according to fish species assemblage and, thus, habitat. Furthermore, census data from sites within marine reserves provide a proxy for quasi-historical conditions (*i.e*., before anthropogenic disturbance) and facilitate production comparisons between modern degraded reefs and historical ‘pristine’ reefs. The outcomes of this work are considered with respect to their implications for the fate and sedimentary significance of fish-derived carbonates in shallow-water regions of tropical and sub-tropical carbonate provinces, and their potential relevance to the inorganic carbon cycle in wider ocean settings.

## Results

### Phase characterisation and relative abundances

**Figure 1:**
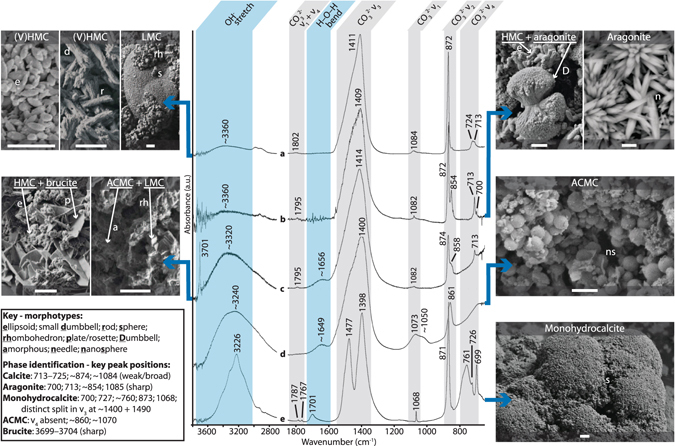
ATR-FTIR spectra and morphological properties for the main forms of fish-derived carbonate. Spectra indicate the following phases: a–calcite (as produced here by *Lutjanus apodus*); b–calcite + aragonite (as produced by *Sparisoma chrysopterum*); c–calcite + ACMC, hydrated + brucite (as produced by *Platybelone argalus*); d–ACMC, hydrated (as produced by *Albula vulpes*); e–monohydrocalcite (as produced by *Sphoeroides testudineus*). Spectra for any given phase are generally similar among species. Vertical bands through spectra: grey = absorption bands associated with carbonate phases, where ν_1_–ν_4_ represent different vibrational modes in the CO_3_^2−^ ion; blue = absorption bands associated with hydrated (ACMC and monohydrocalcite) and hydroxide (brucite) phases, where vibrations are assigned to O-H stretching and H-O-H bending. SEM images show typical morphotypes for each phase; letter symbols indicating morphotype (see key). Scale bars (bottom centre of each image) represent 2 µm.

ATR-FTIR spectra indicate that carbonates excreted by most of the 22 Caribbean fish species sampled are dominated by calcitic phases, with those from some species also generating minor aragonite peaks (Fig. 1). These results are in good agreement with those from previous XRD analyses^12, 13^. However, the additional presence of ACMC, in some cases as the dominant phase, is confirmed for samples from seven species, as indicated by distinctive ν_1_ and ν_2_ peak positions, broader and weaker ν_2_ peaks than those generated by crystalline phases, and the absence of well-defined ν_4_ peaks (Fig. 1–spectrum d). Monohydrocalcite (CaCO_3_·H_2_O) and brucite (Mg[OH]_2_)–phases not previously reported as being produced by fish–are also identified (Fig. 1–spectra c, e), albeit as minor components (<1.5% of total excreted precipitates).

In addition to phase identifications and assignments, FTIR data yield information on several other aspects of fish-derived carbonates that may provide useful insights to precipitation processes and post-excretion fates. Firstly, strong hydration peaks are generated by all fish-derived carbonates containing ACMC (Fig. 1). These spectra, consistent with those generated elsewhere for fish-derived ACMC^22^, indicate that it is a strongly hydrated phase. Hydration peaks are also generated by samples in which (V)HMC is dominant, albeit they are much weaker than those associated with ACMC and monohydrocalcite (Fig. 1). However, they are nearly always stronger than equivalent peaks generated by other biogenic calcites and aragonites we have analysed (Supplementary Fig. 1). Since hydrated amorphous and crystalline carbonates are thought likely to be more soluble than their anhydrous forms^23, 25^, these findings may be of some significance to understanding their post-excretion fates.

Secondly, Politi *et al*.^26^ used the ratio of the intensities between carbonate ν_2_ and ν_4_ absorption bands (out-of-plane and in-plane bending modes for the CO_3_^2−^ ion, respectively; Fig. 1) as a proxy for crystallinity in calcites; lower values being indicative of higher degrees of crystallinity. This ratio is consistently higher in fish-derived (V)HMC ellipsoids (range 7.4–13.2) than in sedimentary HMC foraminifera tests (range 4.2–5.0; Supplementary Fig. 1), which suggests they have a low degree of crystallinity compared to at least some sedimentary HMC and implies that they may be less resistant to dissolution^27^. Ascertaining the relative degree of crystallinity in other fish-derived calcites, however, is hampered by overlapping FTIR peaks resulting from the presence of multiple calcite phases.

Estimates of phase abundance by family, based on SEM assessments informed by these new FTIR data coupled with single-crystal compositional data and existing XRD data^12, 13^, are presented in Table 1, with complete estimates for phases and morphotypes by species presented in Supplementary Tables 1 and 2. Precipitates from eight teleost families (of 15 investigated) are dominated by HMC and VHMC, with those from a further four families being dominated by LMC, and two families by ACMC. The latter phase also accounts for ≥10% of precipitates excreted by fishes from four other families. Of the subsidiary phases, aragonite is the most abundant, being produced by 12 of 15 families and frequently accounting for 5–10% of excreted products. Conversely, monohydrocalcite is produced by only three families, whereas brucite, although present in the carbonates produced by most families, typically accounts for ≪1% of products. Averaging these abundance estimates yields an ‘other family’ category (Table 1), which effectively represents the average phase proportions for all fish families. These proportions were applied to all families seen during fish surveys for which carbonates have not been characterised.

**Table 1:** Estimated volumetric abundances of the various precipitate phases produced by 22 fish species from 15 families

Family*	n^^^	Morphotype abundance (estimated volumetric %)								
		Mg calcite (mol% MgCO_3_)					Aragonite	Monohydro-calcite	ACMC	Brucite
		0–5	5–10	10–15	15–25	>25				
Lutjanidae	2	—	—	—	5	93	2	—	—	—
Serranidae	4	—	—	—	12.25	86.25	1	—	0.5	—
Haemulidae	2	—	—	—	50	50	—	—	—	—
Bothidae	1	—	—	—	—	100	—	—	—	—
Scorpaenidae	1	—	—	—	—	90	—	—	—	10
Sphyraenidae	1	3	—	—	57	—	10	—	30	—
Gerreidae	2	2.5	7.5	—	75	—	10	—	5	—
Belonidae	1	10	—	65	—	—	10	—	10	5
Pomacanthidae	1	95	—	—	—	—	5	—	—	—
Ostraciidae	1	95	—	—	—	—	5	—	—	—
Tetraodontidae	1	75	-	5	—	—	5	10	5	—
Scaridae	1	55	—	—	—	—	30	5	10	—
Labridae	2	30	—	—	—	—	5	—	65	—
Pomacentridae	1	30	—	—	—	—	5	—	65	—
Albulidae	1	—	—	20	30	—	10	5	35	—
*Other* ^†^		*26.4*	*0.5*	*6.0*	*15.3*	*28.0*	*6.5*	*1.3*	*15.0*	*1.0*

*See methodology for details regarding the application of data from each fish species across entire fish families. ^^^n = number of species tested. ^†^Represents all families commonly occurring in The Bahamas for which carbonates are not characterised in this study; values are the average abundances of each phase from all tested families.

### Production modelling

#### Platform-wide production in generalised habitats

**Figure 2:**
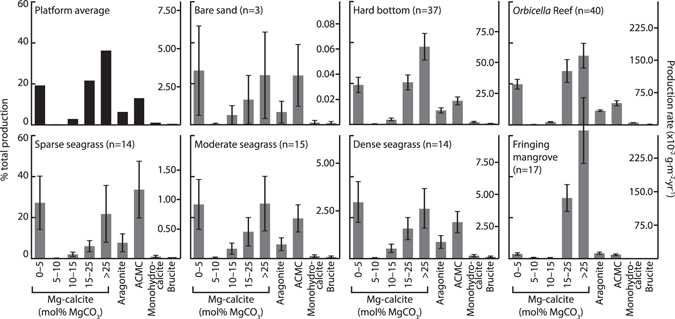
Phase proportions modelled for different habitat types. Fish carbonate phases shown as a proportion of total production in each of seven generalised habitats within The Bahamas, and as a proportion of area-weighted average production across the entire shallow platform area. Right-hand axes show mean (±s.e.m.) production rates. For each habitat type, n is the number of fish biomass survey sites. Data from *Orbicella* reefs are used to represent phase proportions for generalised reefal habitats, although proportions from *Acropora* reefs and patch reefs may be somewhat different (see Fig. 4).

Production values per habitat type (Table 2) show that carbonate production by fish is highest (per unit area) in reefal, hard bottom, and fringing mangrove habitats; production patterns being generally similar to those of Perry *et al*.^12^, but with considerably higher values reflecting the application of an activity scaling factor (after Wilson *et al*.^1^) to yield more realistic outputs. Otherwise, differences that reflect our updating of the production rate–fish body mass relationship (after Perry *et al*.^12^), and the inclusion of additional census data from reefal and hard bottom sites, are minor. However, incorporating our family-specific phase abundance data (Table 1) within this production model provides the first estimates for the total volumes of each phase produced in these habitats, and across the entire platform. Outputs indicate that HMC (specifically that with >15 mol% MgCO_3_) and VHMC–hereafter collectively referred to as (V)HMC–are the dominant phases produced platform-wide (~57% of total production), but also that LMC and ACMC account for significant proportions (~19 and ~13%, respectively). Other phases, however, are generally scarce; platform-wide production totals comprising ~6% aragonite, ~3% HMC (5–15 mol% MgCO_3_), and <1.5% each of monohydrocalcite and brucite (Table 2 and Fig. 2).

**Table 2:** Carbonate production (10^5^ kg·yr^−1^) by fish in shallow-water habitats of The Bahamas.

Habitat and *sub-habitat* type	Area (10^3^ km^2^)	**Total annual production (10** ^**5**^ ** kg)**										Rate* (g·m^−2^·yr^−1^)
		Mg calcite (mol% MgCO_3_)					Aragonite	ACMC	Monohydro-calcite	Brucite	**Total**	
		0–5	5–10	10–15	15–25	>25						
Reef	0.35	3.90	0.03	0.38	7.47	6.06	1.45	2.05	0.24	0.06	**21.65**	**6.19**
*Acropora*	n/m											*7.32*
*Patch reef*	n/m											*9.68*
*Sp/Gr* ^*1*^	n/m											*6.48*
*Orbicella*	n/m											*4.76*
Hard bottom	2.63	8.35	0.08	1.00	8.93	16.45	2.96	4.99	0.47	0.17	**43.40**	**1.65**
*Algal/gorg* *.* ^2^	n/m											*1.65*
*Sargass* ^*3*^	n/m											*1.39*
*Batoph* ^*4*^	n/m											*0.06*
*Complex* ^*5*^	n/m											*16.68*
*Uncolon* ^*6*^	n/m											*1.09*
Fring Mgv^7^	0.54	0.56	0.12	0.08	7.28	15.37	0.65	0.47	0.03	0.01	**24.56**	**4.59**
Sprs s/g^8^	14.42	1.37	0.01	0.10	0.30	1.09	0.39	1.69	0.05	0.02	**5.02**	**0.03**
Md-dty s/g^9^	27.33	7.74	0.12	1.41	3.84	7.89	2.03	5.76	0.37	0.23	**29.39**	**0.11**
Dense s/g	11.43	5.02	0.07	0.89	2.67	4.44	1.48	3.25	0.27	0.15	**18.23**	**0.16**
Bare sand	54.88	0.23	<0.01	0.04	0.11	0.21	0.05	0.21	0.01	0.01	**0.88**	**<0.01**
Platform totals	111.58	**27.18**	**0.43**	**3.89**	**30.61**	**51.51**	**9.02**	**18.41**	**1.43**	**0.65**	**143.13**	

Habitat abbreviations: *Reefal habitats*–^1^Spur-and-groove forereef; *Hard bottom habitats*–^2^Macroalgal/gorgonian plain; ^3^ *Sargassum*-dominated; ^4^ *Batophora*-dominated; ^5^High relief hard bottom with holes and undercut ledges; ^6^Uncolonised pavement with sparse gorgonians; *Other habitats*–^7^fringing mangrove; ^8^sparse and ^9^medium-density seagrass. See Supplementary Table 3 for habitat classification scheme. *Where the main habitat types comprise two or more sub-habitats (*i.e*., reefs and hard bottoms), production rates per unit area are taken as the average of production rates estimated from the most spatially extensive sub-habitats (these are underscored). The overall platform production rate per unit area is taken as the area-weighted average from all the main habitats. n/m = areal extent not mapped.

**Figure 3:**
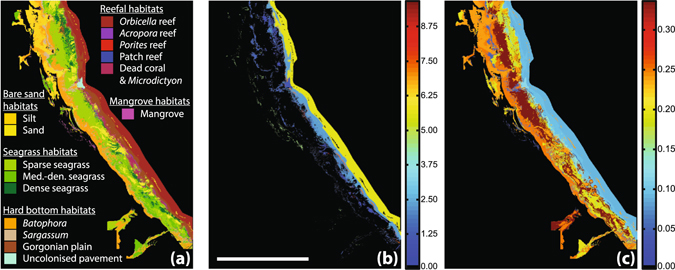
Mapping of production model results. Panels show a section of the shallow platform region adjacent to the eastern shoreline of Andros Island, The Bahamas, detailing: (**a**) habitat distribution; (**b**) overall fish carbonate production rates (g·m^−2^·yr^−1^); and (**c**) production of ACMC as a proportion of total fish carbonate production in each habitat. Note that wide expanses of habitat in which ACMC accounts for >20% of total production are those in which overall production rate is very low. Scale bar in panel (b) represents 10 km. Maps were created by the authors in the software package ERDAS Imagine 8.3 (http://www.hexagongeospatial.com/products/producer-suite/erdas-imagine).

Modelled outputs further indicate that differences in fish densities and species assemblage among habitats result in substantial differences in the relative proportions of carbonate phases produced (Figs 2 and 3). These differences are most striking when comparing (V)HMC with ACMC; reefal and hard bottom habitats tend to yield high proportions of (V)HMC (~58–63% combined production), and low proportions of ACMC (~9–12%), whereas substrates dominated by bare sand or seagrass yield relatively lower proportions of (V)HMC (~28–40%) and higher proportions of ACMC (~18–34%). Likewise, bare sand and seagrass meadows tend to yield higher proportions of LMC (~26–28%) than reefal and hard bottom habitats (~18–19%). Production in fringing mangroves is disparate from all of these habitats; ~92% of total production comprising (V)HMC, with other phases each contributing <3%.

#### Sub-habitat comparisons and marine reserve effects

**Figure 4:**
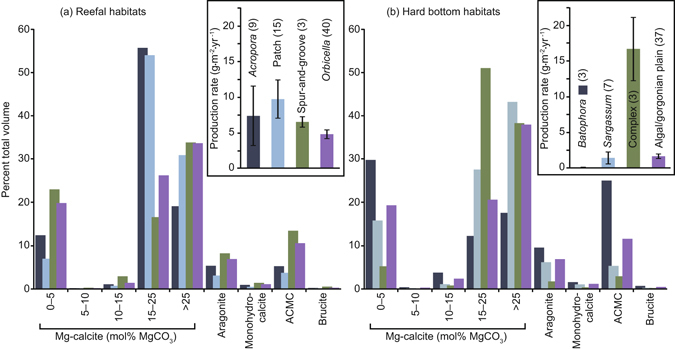
Production charts for sub-habitats within reefal and hard bottom habitat types. Insets show production rates (±s.e.m.) by fish in discrete habitats of (**a**) ‘reefal’ and (**b**) ‘hard bottom’ habitat types. Main charts show the different phases as a proportion of total production in each habitat type. Shading in each of the main charts indicates sub-habitat types as delineated in the corresponding insets. Numbers in parentheses (insets) refer to the number of fish biomass surveys conducted in each habitat type.

Within broad habitat types (Table 2), carbonate production by fishes varies among sub-habitats defined using a higher-resolution classification scheme (Supplementary Tables 3). For example, the relatively coral-rich habitats forming generalised ‘reef’ habitats–*Orbicella* sp. (formerly *Montastraea*) reefs, *Acropora* sp. reefs, and spur-and-groove forereefs–yield production rate means spanning 4.8–7.3 g·m^−2^·yr^−1^ (Table 2 and Fig. 4). In contrast, patch reefs, which are abundant in The Bahamas but omitted from ‘reef’ habitat areal extent estimates, return a higher mean rate of 9.7 g·m^−2^·yr^−1^; a consequence of the concentration of fish biomass into small reef patches surrounded by lagoonal habitats. Generalised ‘hard bottom’ habitats (macroalgal and gorgonian plains) yield mean production rates of 1.7 g·m^−2^·yr^−1^ (Fig. 4), whereas less common hard bottom habitats typically yield lower rates (mean 0.1–1.4 g·m^−2^·yr^−1^; Table 2); only hard bottom areas characterised by relatively high habitat structure return a higher mean production rate (16.7 g·m^−2^·yr^−1^). As with generalised habitats, differences in fish community structure among these sub-habitats yield different proportions of carbonate phases (Fig. 4). For example, ACMC is generated in considerably higher proportions on *Orbicella* reefs and spur-and-groove forereefs (~11–13%) than on patch reefs and *Acropora* reefs (~4–5%).

A critical caveat to these outputs is that they derive from unprotected areas. Thus, fishing pressure has almost certainly diminished overall production rates and potentially, through selective fish extraction, changed natural ratios of phase productivity. To test the impact of fishing pressure we compared production values from unprotected habitats with those from corresponding habitats (specifically, *Orbicella* reefs and macroalgal/gorgonian plains) in a no-take marine reserve (Exuma Cays Land and Sea Park; ECLSP); outputs from the latter being taken as a proxy for historical production in unfished (more ‘pristine’) habitats. Unsurprisingly, removal of fishing pressure (enforced by warden patrols since 1986) has resulted in significantly higher fish species diversity on *Orbicella* reefs in the ECLSP, which support a significantly higher biomass of commercially important large-bodied serranids (23.75 g·m^−2^ inside ECLSP vs. up to 7.93 outside)^28, 29^. Additionally, higher biomass of large serranids and lutjanids in ECLSP hard bottom habitats is a potential reserve effect^29^.

**Figure 5:**
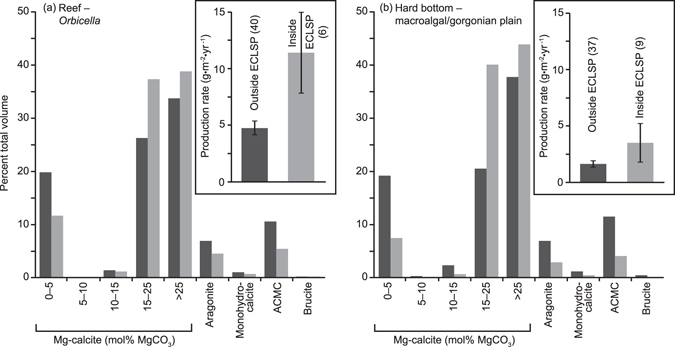
Marine reserve effects on fish carbonate production. Insets show production rates (±s.e.m.) by fish in (**a**) ‘reefal’ and (**b**) ‘hard bottom’ habitat types inside and outside of the Exuma Cays Land and Sea Park (ECLSP). In both habitat types the difference between sites inside and outside ECLSP is significant (one-tailed Mann-Whitney: *P* < 0.05). Main charts show the different phases as a proportion of total production in each habitat type. Shading in each of the main charts corresponds with that shown in each of the insets. Numbers in parentheses (insets) refer to the number of fish biomass surveys conducted within each habitat type.

The impact of these effects on fish carbonate production (Fig. 5) is significant: mean production rates are ~140% higher on *Orbicella* reefs inside the ECLSP than outside (11.40 g·m^−2^·yr^−1^ vs. 4.75; one-tailed Mann-Whitney: *P* = 0.009), and 109% higher on macroalgal/gorgonian plains (3.45 g·m^−2^·yr^−1^ vs. 1.65; one-tailed Mann-Whitney: *P* = 0.033). If comparable effects are applicable across all reefal habitat types, production rates for generalised reefs (6.19 g·m^−2^·yr^−1^ in unprotected areas; Table 2) could be as high as 14.85 g·m^−2^·yr^−1^. These results suggest that removal of fishing pressure from reefal and hard bottom habitats across the entire platform could yield production outputs 54% higher than current models indicate (22.1 × 10^6^ kg·yr^−1^ vs. 14.3 × 10^6^; Table 2), and this value could be even higher if similar effects apply for other habitats. However, effects are probably most pronounced in reefal and hard bottom habitats because these are favoured by many commercially important fishes^30^, biomass increases of which are one of the greatest benefits of no-take reserves^29, 31^.

Comparison of data from corresponding habitats inside and outside the ECLSP further indicate major differences in the proportions of phases produced. Specifically, (V)HMC is produced in higher proportions within the ECLSP for both reefal (76.2 vs. 60.0%) and hard bottom (84.4 vs. 58.5%) habitats, with correspondingly lower proportions of LMC, aragonite, and ACMC (Fig. 5). These differences reflect greater abundance and biomass of commercially important (V)HMC-producing families (*e.g*., Serranidae) within the reserve; selective removal of these families outside the reserve having driven a deviation from normal natural phase proportions. Thus, phase proportions produced over historical timescales will likely have been somewhat different to those modelled for present-day Bahamian settings; a finding that should be used to inform future fisheries management decisions.

## Discussion

New analysis of fish-derived carbonates using FTIR spectroscopy identified a wide range of carbonate and associated phases produced by 22 Caribbean marine fish species. Whilst several of these phases (LMC, HMC, VHMC, aragonite, and ACMC) were identified as common precipitates in previous studies^12, 13, 22^, these new spectroscopic data coupled with novel phase proportion estimates reveal a hitherto unrecognised significance of ACMC and LMC; both are produced in unexpectedly high proportions, and ACMC is produced by more species than previously thought. Monohydrocalcite and brucite are also recognised for the first time in fish-derived precipitates, albeit typically as minor phases (Table 2). Integration of these new data within production models for shallow habitats in The Bahamas generates platform-wide phase quantifications that yield high proportions of LMC, (V)HMC, and ACMC, and low proportions of aragonite, monohydrocalcite, and brucite. In addition, these models allow us to explore among-habitat variations in phase proportions as a consequence of differences in fish species assemblage.

Based on this model, sedimentologists assessing the genetic origins of sediments in contemporary tropical and sub-tropical shallow platform settings might expect evidence for fish-derived carbonates to differ according to depositional habitat (Figs 2 and 3; Table 2). However, several caveats apply: i) evidence for fish carbonates in modern sediments may be obscured by rapid post-depositional alteration or dissolution^13^–a subject of ongoing research; and ii) distribution patterns may be modified by post-excretion sediment transport mechanisms, which may preferentially remove mud-sized (<63 µm) sediments (including most fish carbonates^14^) from higher-energy production sites (*i.e*., reefal and hard bottom habitats), ultimately transporting them either to mud-dominated sediments of lower-energy platform interiors^32^, or to deeper waters off-platform^14, 33^. These uncertainties aside, understanding patterns of fish carbonate production among different fish communities is important for predicting their overall contribution to mud-sized sediment production in tropical and sub-tropical shallow-water carbonate platforms. For example, high production rates associated with reefal, hard bottom, and mangrove habitats means that, even though their collective areal extent covers only 3.2% of shallow platform area of The Bahamas, they generate 63% of the platform-wide fish carbonate production budget (Table 2), resulting in a platform-wide phase proportion signature dominated by (V)HMC (Fig. 2).

The significance of these findings is perhaps best highlighted by extending them across shallow warm-water carbonate provinces beyond The Bahamas, on the assumption that similar production rates and phase proportions apply (albeit with the caveat that fish community structure and biomass are likely to vary among these provinces). This is because the habitat configuration of the Bahamian platform is actually somewhat atypical among shallow warm-water carbonate provinces, particularly with respect to habitats that support high fish biomass concentrations (reefs, hard bottoms, and fringing mangroves): whereas these account for only 3.2% of the total shallow platform area in The Bahamas, they account for 22.9% in the Florida Keys; this being the next smallest proportion in our assessment of other provinces (Table 3). Across other provinces these habitats occupy even higher proportions of the shallow water area, reaching up to 86% at the Northern Line Islands site of Palmyra Atoll. Accordingly, we would predict that the significance of fish carbonate mud to platform-wide production totals is likely to be considerably greater in many of these shallow-water carbonate provinces than in The Bahamas.

**Table 3:** Fish carbonate contributions to platform-wide mud production as a function of benthic habitat configuration

Carbonate province	Areal extent of main generalised habitat categories (% mapped area)^a^					Total fish CaCO_3_ production (overfished system)^b^		Total fish CaCO_3_ production (unfished system)^c^	
	Reef	Hard bottom	Man-grove	Bare sand	Sea-grass	Rate (g·m^−2^·yr^−1^)	% total mud^d^	Rate (g·m^−2^·yr^−1^)	% total mud^d^
The Bahamas	0.3	2.4	0.5	49.2	47.7	0.13	0.2	0.20	0.3
Florida Keys	4.1	18.8	n/r	38.6*	38.6*	0.61	1.3	1.30	2.7
Red Sea (Saudi Arabia)	12.6	14.1	0.3	72.1	1.0	1.03	5.6	2.37	12.2
Puerto Rico	10.4	37.1	4.5	9.0	39.0	1.50	2.9	3.07	5.8
Palau	32.0	28.4	n/r	19.8*	19.8*	2.47	5.0	5.75	10.9
Main Hawaiian Isl.	8.3	61.6	n/r	15.1*	15.1*	1.55	5.8	3.37	11.8
Northern Mariana Isl.	18.3	56.0	n/r	12.9*	12.9*	2.07	6.5	4.66	13.5
Palmyra Atoll	49.8	36.0	n/r	7.1*	7.1*	3.68	7.3	8.64	15.7

a–Benthic habitat data for The Bahamas are delineated in Table 2. Data for the Red Sea are from Bruckner *et al*.^62^ and data for all other regions are from Monaco *et al*.^63^. *‘Unconsolidated sediments’ in some regions were mapped without resolving for bare sand and seagrass habitats. Whilst the ratio of these habitats has little effect on platform-averaged fish carbonate production rates (because rates are relatively low in both), it has a large effect on estimates of % contribution to total mud production–because rates for sources other than fish are low in bare sand habitats and high in seagrass habitats^12^ (see footnote d). Thus, ‘unconsolidated sediments’ not resolved for specific habitats are cautiously assumed to comprise bare sand and medium-density seagrass at a 1:1 ratio. Areal data were not resolved (n/r) for mangrove habitats in some regions. b–Platform-averaged production rates in over-fished systems are estimated for each province using habitat-specific production rates for The Bahamas (Table 2), with the caveat that fish biomass and carbonate production is likely to vary among these regions. Production rates for medium density seagrass are used where densities are not reported. c–Platform-averaged production rates in unfished systems are estimated following application of scaling factors to generalised reefal and hard bottom habitat production rates (140% and 109% respectively, based on data from a no-take marine reserve; Fig. 5). Scaling factors for other habitats are assumed to be zero because they support fewer commercially important fishes^30^, and effects of fishing pressures are therefore likely to be less pronounced in these habitats. d–Production rates for carbonate mud from sources other than fish are estimated for each province using a habitat-specific mud production budget constructed for The Bahamas^12^. As such, the fish carbonate contributions to total mud production are expressed here with the caveat that actual mud production budgets are likely to vary by region.

To test this prediction, we applied our habitat-specific production rate estimates from The Bahamas across a number of other global localities (Table 3), with inherent caveats as stated above. This current best-estimate approach suggests that platform-averaged production rates from test regions are typically in the range 0.61–3.68 g·m^−2^·yr^−1^, compared with 0.13 g·m^−2^·yr^−1^ in The Bahamas. If a Bahamian mud production budget^12^ involving other carbonate sources is also broadly applicable across these provinces, fish carbonates will typically account for 1.3–7.3% of total carbonate mud production, compared with 0.2% in The Bahamas. Given the magnitude of these differences in production totals, one might also expect these contrasting habitat configurations to yield different platform-wide phase proportions. However, as alluded to above, platform-wide phase proportions in The Bahamas are effectively an average of phase proportions generated in reefs and hard bottoms (Fig. 2); such is the dominance of these habitats in terms of fish carbonate generation there. Consequently, increasing their proportional extent, even dramatically, has only a small effect on platform-wide phase proportions, typically manifested as (V)HMC totals up to ~62% (cf. 57% in The Bahamas) and ACMC totals down to ~10% (cf. 13% in The Bahamas).

It is important to bear in mind that these calculations are based on model outputs for overfished settings. Adjusting habitat-specific rates in our test regions to those estimated from our marine reserve data (Fig. 5), we find that platform-averaged rates approximating fish carbonate production over historical (and perhaps geological) timescales in these provinces could have been in the range 1.30–8.64 g·m^−2^·yr^−1^ (2.7–15.7% total carbonate mud production; Table 3). Furthermore, these historical estimates are likely conservative, since data from other regions suggest that the capacity for many no-take marine reserves to restore fish biomass to truly pristine states may be limited by their restricted size and proximity to overfished areas. Indeed, the highest documented biomass concentrations of reef-associated fishes are from large and isolated marine reserves (*e.g*., the Chagos Archipelago) that perhaps best approximate true ‘wilderness’ settings^34^ and could potentially yield fish carbonate production rates far higher than those estimated here.

A major implication of the diverse range of fish carbonate phases is that they likely have differing post-excretion fates. This is a particularly important outcome since it can provide insights to the nature of fish carbonate contribution to sedimentary processes in shallow water settings through consideration of the relative abundance and solubility potential of each phase. Solubility measurements involving VHMC ellipsoids produced by the gulf toadfish (*Opsanus beta*) indicate they are nearly twice as soluble as aragonite, with results being broadly comparable with other types of sedimentary HMC^8^. It is therefore reasonable to assume that fish-derived precipitates have solubilities comparable with those of similar phases precipitated via other biogenic (where data are available) or abiotic processes, although factors such as the low degree of crystallinity, relatively higher water contents, and small crystal size of some fish-derived carbonates (*e.g*., VHMC ellipsoids), may further influence their solubilities^19, 27^. Whilst this approach is complicated by considerable disparity among published Mg-calcite solubility relationships^19^, we follow the approach of Morse *et al*.^35^ by considering two relationships for Mg-calcites: (1) the ‘best fit’ biogenic relationship^17, 18^; and (2) the ‘Plummer and Mackenzie’ relationship^36, 37^.

**Figure 6:**
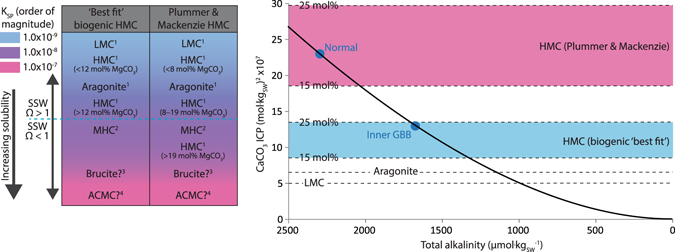
Likely solubility orders of fish carbonate phases and their theoretical fates in different surface seawater scenarios in The Bahamas. Solubility orders (left) are based on existing data for similar phases of biogenic (where available) and synthetic origins. Differences between the columns reflect a disparity in published solubility data for HMC. Data are from: 1) Morse and Mackenzie^19^ (and references therein); 2) Hull and Turnball^64^; 3) Walter and Morse^17^; 4) Brečević and Nielsen^23^ and Clarkson *et al*.^65^. Phases below the dashed line are thermodynamically unstable (Ω < 1) under normal surface seawater (SSW) conditions in The Bahamas. Based on these solubility data, a plot of CaCO_3_ ion concentration product (ICP; *i.e*. [Ca^2+^][CO_3_^2−^]) for seawater (practical salinity 35; temperature 25 °C; ρCO_2_ 400 µatm) versus total alkalinity illustrates the predicted stabilities of these phases relative to SSW in The Bahamas (right; modified after Morse *et al*.^35^ with the kind permission of Elsevier). ICP values for normal and platform interior (Inner GBB) waters are plotted (solid circles) along with equilibrium ICP values for relevant carbonate phases (dashed lines; Ω = 1); these phases being more stable the further their equilibrium ICP values are below seawater ICP. Uncertainties regarding brucite and ACMC solubilities are discussed in Supplementary Note 1, but available data suggest both phases are highly unstable in SSW.

On the basis of solubility orders determined using these relationships (Fig. 6), the post-excretion fates of fish-derived carbonates in surface seawater can be predicted if DIC-system parameters, and thus CaCO_3_ saturation states (Ω), are known. ‘Normal’ surface seawater around The Bahamas is characterised by elevated alkalinity^38^, with Ω_calcite_ ≈ 6 and Ω_aragonite_ ≈ 4, whereas poorly circulating waters overlying platform interiors can have depleted alkalinities and correspondingly lower saturation states (Ω_calcite_ ≈ 4 and Ω_aragonite_ ≈ 2)^39^. Thus, predicting the fate of some fish carbonate phases is straightforward: LMC and aragonite should be stable under all surface seawater scenarios in The Bahamas, whereas monohydrocalcite, ACMC, and brucite should be unstable. Of these unstable phases, monohydrocalcite probably dehydrates and transforms to more stable phases under surface seawater conditions^40, 41^. Amorphous carbonates can undergo similar transformations^25^, although available evidence suggests that fish-derived ACMC undergoes rapid (hours to days) dissolution^22^, whilst brucite likely follows a similar fate^17, 42^.

The fate of (V)HMC, however, is more problematic to predict, in part because of uncertainty regarding solubility curves, but also because their fates will vary depending on where they are deposited (Fig. 6). For example, employing the ‘Plummer and Mackenzie’ solubility curve, HMC containing less than ~19 mol% MgCO_3_ should be stable in ‘normal’ seawater, whereas the equivalent threshold composition in poorly circulating platform interior waters could be as low as ~11 mol% MgCO_3_. These compositional thresholds are higher when using the ‘best fit’ biogenic curve, but the same principle applies. In either case, some fish-derived (V)HMC will be thermodynamically unstable at surface seawater conditions, although this does not necessarily mean it will dissolve. Rapid dissolution of reagent-grade calcite^43^ of comparable grain size to fish-derived carbonates requires Ω_calcite_ < 0.8. If a similar rule applies for fish-derived (V)HMC, low dissolution rates in waters where Ω_(V)HMC_ = 0.8–1.0 could be overridden by the rate at which original grains alter to more stable forms, as has been shown to proceed very rapidly (months) in algal-derived HMC^44^. Thus, the fates of fish-derived (V)HMC in these platform settings remain somewhat uncertain, but their high MgCO_3_ contents mean they likely represent some of the least stable carbonate phases in tropical and sub-tropical carbonate platform settings, and are probably ‘early responders’ where dissolution is concerned.

Integration of these predicted fates in our production model yields a first-order estimate of the proportion of fish-derived carbonate that is thermodynamically unstable at surface seawater conditions in The Bahamas. On a platform-wide scale, ~29% of fish-derived carbonate (LMC, aragonite, and HMC with 5–15 mol% MgCO_3_; Table 2) should be stable at surface conditions, whereas ~14% (ACMC and brucite) may rapidly dissolve. The fate of (V)HMC (the remaining ~57%) is less certain: the ‘best fit’ biogenic solubility relationship suggests most will be stable in normal surface seawater, whereas the ‘Plummer and Mackenzie’ relationship suggests most will be thermodynamically unstable (Fig. 6).

**Figure 7:**
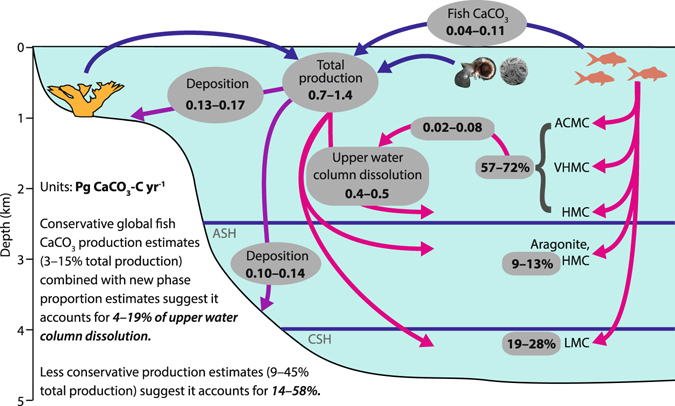
Conceptual model of the fish carbonate contribution to marine inorganic carbon cycling. Arrows represent major sources of CaCO_3_ production (blue); CaCO_3_ deposition as sediment (purple); and CaCO_3_ dissolution (pink). Predicted dissolution depths are for a North Atlantic scenario, where ASH and CSH are aragonite and calcite saturation horizons (Ω = 1), respectively (Feely *et al*.^16^). Assuming phase proportions of total fish CaCO_3_ production reflect those estimated for most shallow-water habitats in The Bahamas, ~57–72% (ACMC and (V)HMC) is predicted to dissolve in the upper water column, accounting for 4–19% of total upper water column dissolution. This contribution will be greater (7–25%) if total production is more similar to that in Bahamian mangrove habitats (94% ACMC and (V)HMC), and even more significant if less conservative estimates of global fish carbonate production are used. Fish carbonate production estimates are from Wilson *et al*.^1^; total carbonate production, deposition, and dissolution estimates are from Feely *et al*.^16^ and Milliman *et al*.^66^. Figure is modified after Wilson *et al*.^1^ with the kind permission of The American Association for the Advancement of Science.

Clearly further experimental work on the preservation and dissolution pathways of fish-derived carbonate phases is required to overcome these theoretical prediction issues, but our findings may nevertheless serve as a guide to further work that will help to constrain the production and cycling of fish-derived carbonates on a wider global scale. In particular, these findings provide strong evidence for a marked phase diversity in fish carbonate products occurring at a local scale, and it is reasonable to speculate that similar patterns may be replicated at the global scale. It is well beyond the scope of this study to attempt to model global scale phase proportions, as that would at least require additional phase production datasets from pelagic and cool-water fish groups–which are currently sparse at best. However, it is worth illustrating the potential implications that phase diversity in fish carbonates could have at the global scale. This is a highly relevant issue because fish have recently, and on the assumption that they produce only (V)HMC, been invoked as having a major role in the cycling of inorganic carbon in the upper 1 km of the oceans^1, 8^. Using our Bahamian dataset to populate an entirely conceptual oceanic model (Fig. 7), however, it is evident that dissolution of fish carbonates potentially occurs over a much greater depth range than initially hypothesised, and that their role in the marine inorganic carbon cycle could be considerably more complex and diverse than previously thought.

Constraining the role of fish carbonates in the wider oceanic setting is particularly important, not only because of the apparent magnitude of their contribution to the inorganic carbon cycle^1^, but because of the potential for this role to change in the future. In part, changes could relate to rising levels of atmospheric CO_2_ and subsequent elevations in surface seawater temperatures and pCO_2_, both of which are predicted to stimulate increased carbonate production rates at the level of individual fish^1^, albeit effects remain to be fully quantified^45^. Such a response would contrast with that predicted for many other calcifying organisms under elevated pCO_2_^46^, and it is possible that fish will contribute an increasingly large proportion of total marine carbonate under future climate change scenarios. However, such a change is potentially being offset by dramatic global declines in fish biomass due to recent and ongoing fishing pressures^47^. Furthermore, associated shifts in fish species composition could, as we see at the local scale here, drive a shift in overall fish carbonate phase proportions.

Our findings thus highlight that, in addition to these potential future changes in the magnitude of the fish carbonate contribution to inorganic carbon cycling, the nature of this contribution remains poorly understood due to a paucity of carbonate phase data, and is subject to change in response to shifting proportions of these carbonate phases. Since the inorganic carbon cycle controls the distribution of alkalinity throughout the oceans^16^, all of these issues could have implications for future climate through their potential effects on the capacity of the surface ocean to buffer increases in atmospheric CO_2_. Thus, there is a pressing need for additional data that will help to quantify phase proportions across a wider range of fish communities in different global marine settings. Models that incorporate such data will then underpin further work aimed at quantifying the role of fish in past, present, and future cycling of inorganic carbon across global scales, potentially with consequences for future fisheries management as a carbon-regulating service^9^.

## Methods

### Carbonate collections

Gut carbonate samples were collected from 22 fish species common to the Caribbean using aquaria facilities at the Cape Eleuthera Institute, Eleuthera Island, The Bahamas. Sampling was conducted in November 2009, July 2010, and in May and December 2011, thus encompassing the full range of seasonal temperature fluctuations. Fish were held in natural seawater at local ambient surface conditions (salinity in the range 36–37 on the practical salinity scale; temperature in the range 25–30 °C); all water having first passed through a 1 µm filter to ensure no external particles could be ingested by the fish. Food was withheld throughout the study to ensure that sample material comprised only gut-produced carbonates. To further facilitate sample purity, carbonate pellets were collected only after fish were held for an initial 48 hour period to ensure their guts were completely voided of other materials. Excreted carbonate pellets were then recovered from tank floors using disposable 10 mL Pasteur pipettes, typically at 24 hr intervals, before being cleaned with sodium hypochlorite (commercial bleach), rinsed with distilled water, and dried at 50 °C, following procedures recommended for the preparation of naturally occurring carbonates^48^. Animal collection and holding was conducted under approval by The Bahamas Department of Marine Resources and in accordance with animal care guidelines set out in UK Home Office Project Licence PPL 30/2735.

Tank floor recovery is a widely adopted approach to sampling piscine gut carbonates^8, 12–14, 22, 45^ but other methods include direct sampling from the intestinal tract (following animal euthanasia)^8, 22, 45^ and collection in a surgically attached rectal sac^45^. Direct sampling from the intestine was unsuitable here for two reasons. Firstly, animal euthanasia precludes the possibility of measuring carbonate production rates. Secondly, the stages involved in gut carbonate precipitation are poorly understood but it is possible that fish carbonates, like many other biogenic carbonates^25^, form via an amorphous precursor. The possibility then arises that precipitates are dominated by ACMC in the foregut but become increasingly more crystalline as they ‘mature’ towards the hindgut, as has been documented in at least one instance elsewhere^22^. Direct intestinal sampling thus has the potential to yield misleading compositional and mineralogical data. The rectal sac approach, which allows excreted solids and fluids to accumulate in isolation from surrounding seawater, is an excellent means of controlling for post-excretion processes affecting precipitates (*e.g*., dissolution) in experiments involving different seawater treatments^22^. However, for the purposes of this study it is problematic because the interval between excretion and retrieval of precipitates effectively represents an artificial lengthening of the time for which they are in contact with gut fluids, thus potentially prolonging precipitation and crystallisation processes. Consequently, this approach might also yield misleading mineralogical and compositional data.

Collection of excreted carbonates from tank floors is therefore the only sampling strategy in which we can be confident that products are representative of those entering the marine environment. A limitation of this approach, however, is that exposure of carbonates to seawater introduces the possibility of sample loss through dissolution; an issue likely to be most pronounced for ACMC, which can dissolve very rapidly in seawater^22^. For this reason, samples thought to contain ACMC (*e.g*., those produced by labrids and pomacentrids) were omitted from production rate determinations. For all other carbonate types (*e.g*., HMC ellipsoids, LMC spheres), we observed no differences in phase proportions or particle properties (morphology, composition, and phase) when comparing freshly excreted samples with those collected within 24 hrs of excretion by the same individual fish. This observation suggests these carbonate types undergo limited or no change within the first 24 hrs of seawater exposure.

It is further necessary to point out that fasting of study animals will have lowered their metabolic rates and potentially affected gut fluid composition, thus raising the possibility that samples collected during the study were not representative of fish carbonates produced under normal natural circumstances. However, gut carbonates excreted alongside faecal matter have been shown to be similar to those produced during periods of fasting^13^. Furthermore, samples produced by fasting barramundi (*Lates calcarifer*) are identical to those produced by the same individuals after a controlled diet of prawn flesh (n = 4) or squid flesh (n = 4) was introduced (Supplementary Fig. 2). Thus, available data suggest that carbonates produced by fasting fish are similar to those produced under normal natural circumstances, although further work on this subject is necessary.

### Carbonate characterisation and phase abundance estimates

ATR-FTIR analyses were performed at a resolution of 2 cm^−1^ using a Nicolet 380 FTIR spectrometer coupled with a Thermo Scientific SMART iTR ATR sampler equipped with a diamond reflecting cell, with final spectra obtained by the co-addition of 32 repeated scans. Analyses were performed on at least three powdered sub-samples (each comprising 2–3 pellets) per species to ensure representative spectra were obtained. Identification of the carbonate phases was achieved through reference to an extensive spectral database (Supplementary Tables 4– 6). Additionally, some samples were analysed using transmission FTIR spectroscopy in order to better resolve peaks at low wavenumbers (<600 cm^−1^). Powdered CaCO_3_ pellets were homogenised with KBr and pressed into transparent discs (~0.5 mm thick) which were then analysed with a Nicolet FTIR spectrometer using analytical settings as described for ATR-FTIR spectroscopy.

Although FTIR spectroscopy is effective at discriminating between discrete carbonate phases, we found it unreliable for determining MgCO_3_ contents in fish-derived carbonates, despite the existence of a published relationship between ν_4_ (in-plane bending mode) peak position and MgCO_3_ content in well crystallised biogenic Mg calcites^49^. It is possible that this relationship does not hold for fish-derived Mg calcites due to their low degree of crystallinity (see Results). Consequently, where spectra indicate the presence of calcite, this may refer to any of LMC, HMC, or VHMC; more specific identification only being possible through acquisition of compositional and/or XRD data.

Samples were characterised with respect to morphology and chemical composition using scanning electron microscopy (SEM) and energy-dispersive X-ray spectroscopy (EDX) within a Zeiss Supra 40VP field emission gun system with integrated Oxford Instruments ISIS EDX detector. A minimum of 10 EDX scans were performed in discrete regions of each of at least 5 (and up to 30) pellets per fish species, incorporating all particle morphotypes known to be produced by each. Because the spatial resolution of these scans exceeds the size of many of the subject particles, scans were only performed if subject particles were surrounded by similar particles, or if they were of sufficient size to overcome this issue. Detailed results concerning MgCO_3_ contents determined using this approach are presented elsewhere^13^. Briefly, variability in MgCO_3_ content within species is most pronounced when comparing different particle morphotypes. For example, sample- and species-specific MgCO_3_ contents are typically higher in micron-scale ellipsoids (*e.g*., typically ~25–35 mol%) than they are in ~20 µm dumbbells (*e.g*., typically ~5–15 mol%). In addition, within some particle morphotypes there can be considerable variability among samples produced by any given fish species (*e.g*., 1 S.D. can be as large as 9 mol% MgCO_3_) and even within samples produced by individual fish, with the greatest variability being seen in ACMC (nanospheres and material lacking any definable morphology) and VHMC (ellipsoids). It is worth mentioning here that controls on Mg^2+^ incorporation in calcites generally can be complex and wide-ranging, but a positive correlation between MgCO_3_ content and temperature is frequently reported^50^. However, we found no significant differences in the MgCO_3_ contents of each particle morphotype when comparing samples obtained in winter (SST ≈ 25 °C) with those obtained from the same fish species in summer (SST ≈ 30 °C), suggesting that seasonal SST fluctuations in The Bahamas are insufficient to influence gut carbonate magnesium contents.

Compositional and morphological data were then combined with FTIR data to facilitate assignment of carbonate phases (*i.e*., calcite categorised by mol% MgCO_3_ content, aragonite, monohydrocalcite, ACMC, or brucite) to each morphotype. In some samples the presence of a single mineral phase (as indicated by FTIR data) meant assignment of phase to morphotype was straightforward. However, many samples comprised multiple phases and it was then necessary to differentiate morphotypes on the basis of magnesium and strontium contents (using morphotype-specific compositional data from EDX spectroscopy) before assigning phases in accordance with published phase–composition relationships^51, 52^. Variability of MgCO_3_ content within morphotypes was generally not problematic in this regard because it was most pronounced in ACMC and VHMC, both of which are defined by a broad range of MgCO_3_ contents (*i.e*., no specified limits in ACMC and >25 mol% in VHMC). Where MgCO_3_ contents did span two or more phase categories (*e.g*., VHMC and HMC with 15–25 mol% MgCO_3_), we apportioned a relative abundance to each based on the average spread of MgCO_3_ contents for the species concerned. This approach was performed for samples produced by 16 fish species, and morphotype phases were generally consistent among these (*e.g*., all monocrystalline ellipsoids were VHMC or HMC). Samples from an additional 6 species were of insufficient size for FTIR and XRD analyses, so phases were assigned using only morphological and compositional data on the assumption that morphotype phases are consistent among all fish species (compositional data support this assumption). Following phase assignments, >30 pellets from each species were examined using SEM to facilitate visual estimation of the volumetric abundances of all morphotypes, and thus phases.

Finally, we extrapolate our abundance estimates across entire fish families by averaging the totals for species within each family tested. The validity of this approach is substantiated by a pattern emerging from published data^13^ and our ongoing work that indicates strong intra-family consistency in precipitation products. For example, of nine lutjanid species we have tested, all predominantly produce (V)HMC ellipsoids, and of eleven labrid species tested, all produce ACMC with subsidiary LMC. Precipitation products are similarly consistent for all families (n = 13) in which multiple species have been tested, these including serranids (n = 10), pomacentrids (n = 3), and scorpaenids (n = 4).

### Production rates and modelling

Production rates were determined using carbonates excreted over known time (typically 24–48 hours) by fish of known mass. The mass of excreted carbonates was determined from cleaned dried samples either by weighing, or by acid-base titration using 0.02 M HCl and 0.02 M NaOH; the amount of acid required to dissolve the entire sample being used to calculate the number of moles of (Ca,Mg)CO_3_ in the original sample. Results were then converted to weight values (accounting for known ratios of MgCO_3_ to CaCO_3_), which typically differed from direct weight measurement values by <2%.

Measured production rates, however, are highly conservative because they are derived from starved and inactive fish (inactivity being a consequence of confinement). The cumulative effect of these experimental conditions will have been to lower metabolic rates and thus ultimately to lower carbonate production rates^1^. Estimates of scaling factors that account for the difference between normal metabolic rates and those depressed through removal of feeding and physical activity are in the range 2.5–3.4^53^; the lowest of these values being employed elsewhere^1^ to conservatively adjust measured fish carbonate production rates to more practical real-world values. We follow this approach in order to generate realistic estimates of fish carbonate production rate in The Bahamas.

Production calibration data were then analysed by regressing production rates against log-transformed values of fish biomass and a quadratic term for log biomass to account for any potential curvilinear relationships. Fish family was included as a random factor, so analyses used mixed-effects linear models in the nlme package^54^ in R statistical software^55^. In addition to the data from fish families measured empirically, the model included an ‘other’ family, parameterised as the mean biomass and production rate of all other families. This additional family category allowed the model to be used to predict production rates by fishes from all other families where species-level data were unavailable. Models were fitted using the procedure outlined by Crawley^56^. Briefly, a maximal model was fitted including all factors. Least significant terms were then removed in turn, and after each term was removed models were compared to ensure that term removal did not lead to a significant increase in deviance or Akaike information criterion (AIC). Terms were removed until the model contained only significant terms or removal of any non-significant terms caused a significant increase in deviance or AIC (minimal adequate model; see Supplementary Methods 1).

Data for fish abundances across The Bahamas are described in detail elsewhere^57^, so are only briefly described here. Data were collected from replicate sites in all major hard- and soft-bottom habitats found around each of nine islands: Abaco, Andros, Bimini, Conception, Eleuthera, Grand Bahama, Lee Stocking, San Salvador, and the Turks and Caicos, and from sites in and around the Exuma Cays Land and Sea Park (ECLSP). Sites were selected from seven generalised habitats of known areal extent, as delineated by Perry *et al*.^12^, of which ‘reefs’ and ‘hard bottoms’ are further sub-divided on the basis of ecology and/or geomorphology–see Supplementary Table 3 for classification scheme. At each site, all but nocturnal (*e.g*., Apogonidae) and cryptic (*e.g*., Clinidae and Gobiidae) fish species were surveyed using discrete group visual fish census^58^. The exclusion of data on cryptic species may mean that our overall rate estimates are somewhat conservative. However, the extent of underestimation may be limited given that small cryptic fish species not seen during visual surveys in a different reef system, although abundant, reportedly account for only 1% of total biomass^59^. Species were divided into three groups and density and size (to nearest cm) estimated along belt transects. Transect size and number was optimized through the use of data from equivalent surveys within the Caribbean^60^.

The calibration model was then used to generate species- or family-specific production rates for every teleost fish seen during field surveys. Firstly, data on fish lengths were converted to biomass using allometric relationships^61^, and these biomasses were used to generate production estimates from the model. The family-specific mean phase proportions (including variations of MgCO_3_ content in calcites) were then used to separate the total carbonate production per fish into production values of each phase per fish. Estimates for the entire fish assemblage seen on each transect were then summed and averaged across transects to produce an estimate of mean production per transect for each fish group. All values were standardised to production per 200 m^2^, and mean production per 200 m^2^ for each of the three fish groups were summed to provide production estimates for the entire assemblage per site. Since multiple sites were surveyed per habitat type, data were averaged to generate mean production rates of each morphotype and phase in each habitat type.

## Electronic supplementary material

Phase heterogeneity in carbonate production by marine fish influences their roles in sediment generation and the inorganic carbon cycle–Supplementary Information
