# Supplementary material for: Phase heterogeneity in carbonate production by marine fish influences their roles in sediment generation and the inorganic carbon cycle
Source: Sci Rep. 2017 Apr 10;7:765. doi: 10.1038/s41598-017-00787-4 (PMC5429630; doi:10.1038/s41598-017-00787-4)
Supplement: Supplementary file 1 — Phase heterogeneity in carbonate production by marine fish influences their roles in sediment generation and the inorganic carbon cycle–Supplementary Information [file 41598_2017_787_MOESM1_ESM.pdf]

Phase heterogeneity in carbonate production by marine fish influences their roles in sediment generation and the inorganic carbon cycle

Michael A. Salter, Alastair R. Harborne, Chris T. Perry, and Rod W. Wilson

Supplementary Information

Supplementary Figure 1: *ATR-FTIR spectra for: (A) gastropod fragments; (B) foraminifera test fragments; and (C) intestinal precipitates excreted by a marine fish (Lutjanus apodus).*

Supplementary Figure 2: *SEM images and FTIR spectra showing similarities between fish carbonates produced during periods of fasting and feeding.*

Supplementary Table 1: *Carbonate (and associated) phases determined using FTIR spectroscopy and EDX spectroscopy in pellets excreted by 22 Caribbean fish species, and the estimated abundances of each per fish species.*

Supplementary Table 2: *The main precipitate morphotypes excreted by 22 Caribbean fish species, their estimated abundances per fish species, and the phases assigned to each based on results of FTIR and EDX spectroscopy.*

Supplementary Table 3: *Habitat classification scheme for Bahamian shallow platform settings.*

Supplementary Table 4: *Infrared spectroscopic data reported in literature for calcite, Mg-calcite, and aragonite.*

Supplementary Table 5: *Reported infrared wavenumbers for hydrous Ca- and Mg-bearing carbonate (and related) phases.*

Supplementary Table 6: *Reported infrared wavenumbers for selected additional anhydrous Ca- and Mg-bearing carbonate phases*

Supplementary Note 1: *Further discussion regarding the fates of brucite and ACMC.*

Supplementary Method 1: *Production–biomass calibration model parameters.*

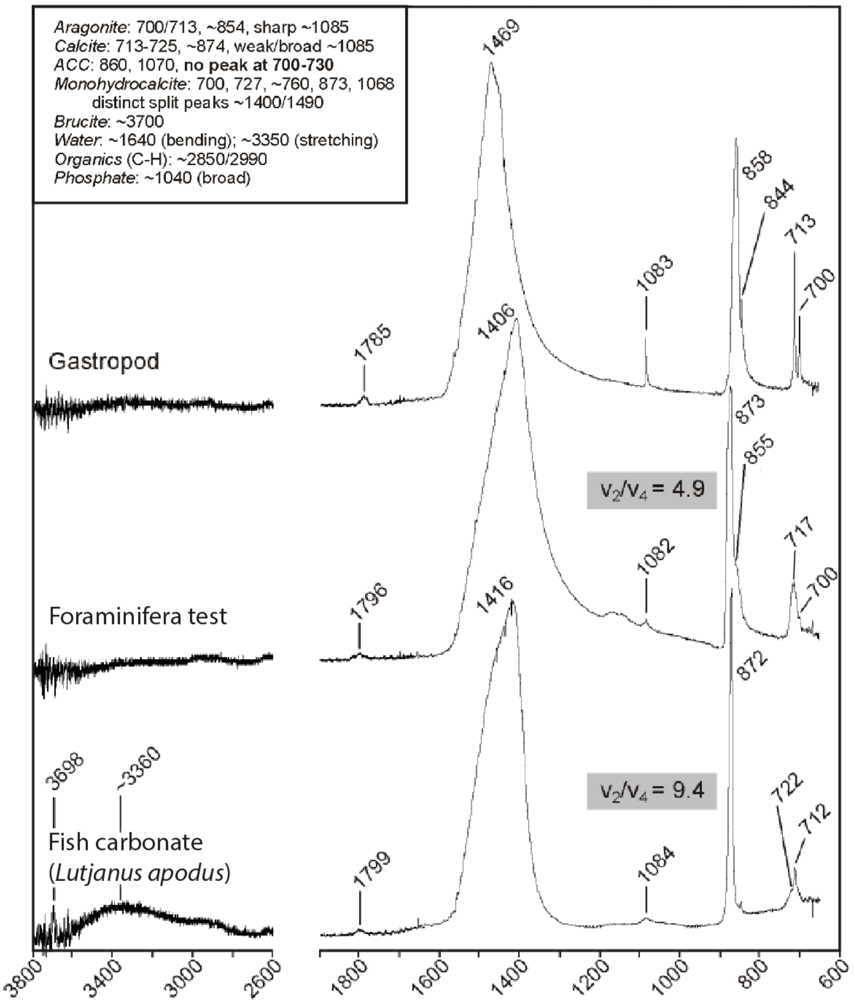

Supplementary Figure 1 *ATR-FTIR spectra for: (A) gastropod fragments; (B) foraminifera test fragments; and (C) intestinal precipitates excreted by a marine fish (Lutjanus apodus)*. Key peak positions for the relevant phases are detailed in the inset box, top left. Left-hand portions of spectra (wavenumbers >2600 cm^{-1}) are 4x vertically exaggerated relative to right-hand portions. Material for spectra (A) and (B) was isolated from surface sediment samples collected from a shallow-water bare sand habitat in Eleuthera Sound, The Bahamas. Spectra show that the gastropod fragments are aragonitic, whereas the foraminifera test fragments are predominantly HMC with subsidiary aragonite. The spectrum for fish carbonate also indicates HMC as the dominant phase. However, whereas both sedimentary carbonate types are anhydrous (evidenced by the absence of OH^- stretching peaks and H-O-H bending peaks at wavenumbers of ~3300 and ~1630, respectively), a weak OH^- stretching peak (at ~3300 cm^{-1}) is generated by the fish carbonate; generation of such peaks being typical for fish-derived HMC, regardless of the presence of brucite (a weak but sharp peak at 3698 cm^{-1} indicates it is present in the sample shown here). Since there is no evidence to suggest that other hydrated phases are present in such samples, these spectra suggest that fish-derived HMC is weakly hydrated. Note also the ratio of v_2 and v_4 peak heights for fish-derived HMC (9.4; range 7.4–13.2 in all samples tested from 9 fish species), which is always higher than that for foraminiferal HMC (4.9; range 4.2–5.0) and indicates a relatively low degree of crystallinity in the former (this was only be tested for HMC ellipsoids – inherent issues with other sample types, such as overlapping peaks, precluding similar analyses).

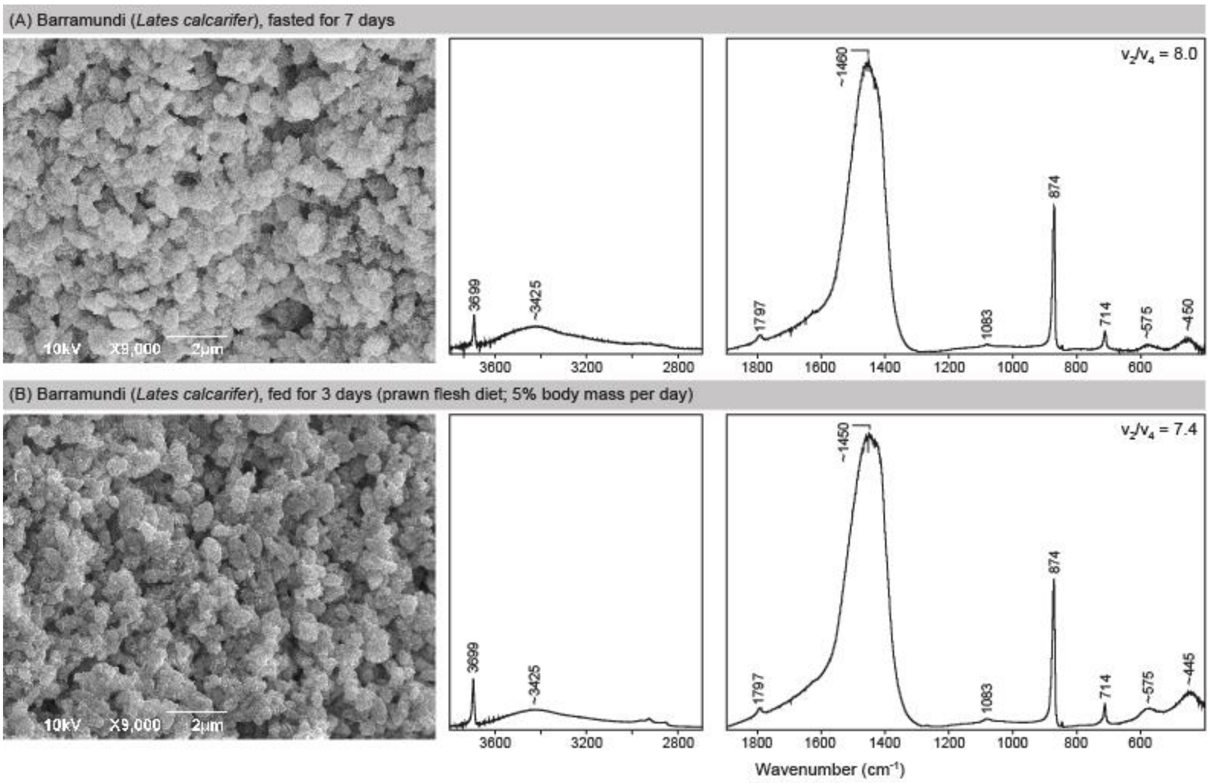

Supplementary Figure 2 *SEM images and transmission FTIR spectra showing similarities between fish carbonates produced during periods of fasting and feeding.* SEM images show that carbonates produced by an individual barramundi (*Lates calcarifer*) during periods of fasting (A) and feeding (B) are morphologically similar; dominated in both cases by micron-sized ellipsoids, many of which are partially coated with plate-shaped precipitates. FTIR spectra indicate that calcite (ellipsoids) is the dominant phase produced in both cases, with brucite (plate-shaped precipitates) occurring as a subsidiary phase (see Supplementary Figure 1 for key to peak positions). Ratios of peak intensities between v_2 and v_4 are also similar in both cases, suggesting that degree of crystallinity is not influenced by feeding state. Similar results to these were obtained for all tested individuals of barramundi (n=8), of which 4 were fed a diet of prawn flesh (abdominal segments; hard parts removed), and another 4 were fed a diet of squid mantle flesh, each at a rate of 5% body mass per day. Left-hand portions of FTIR spectra (wavenumbers >2600 cm^{-1}) are 4x vertically exaggerated relative to right-hand portions.

Supplementary Table 1 Carbonate (and associated) phases determined using FTIR spectroscopy and EDX spectroscopy in pellets excreted by 22 Caribbean fish species, and the estimated abundances of each per fish species.

		**Precipitate phase[a] volumetric abundance (%)[b]**								
		Mg-calcite (mol% $MgCO_3$)								
**Fish species**	**Family**	0–5	5–10	10–15	15–25	>25	Aragonite	Mono-hydrocalcite	ACMC	Brucite
Albula vulpes	Albulidae	0	0	**20**	**30**	0	**10**	**5**	**35**	0
Platybelone argalus argalus	Belonidae	**10**	0	**65**	0	0	**10**	0	**10**	**5**
Bothus sp.*	Bothidae	0	0	0	0	**100**	0	0	0	0
Eucinostomus sp.*	Gerreidae	0	0	0	**80**	0	**10**	0	**10**	0
Gerres cinereus	Gerreidae	**5**	**15**	0	**70**	0	**10**	0	0	0
Haemulon flavolineatum	Haemulidae	0	0	0	**100**	0	0	0	0	0
Haemulon sp.*	Haemulidae	0	0	0	0	**100**	0	0	0	0
Halichoeres bivittatus	Labridae	**30**	0	0	0	0	**5**	0	**65**	0
Thalassoma bifasciatum	Labridae	**30**	0	0	0	0	**5**	0	**65**	0
Lutjanus apodus	Lutjanidae	0	0	0	**10**	**88**	**2**	0	0	0
Ocyurus chrysurus	Lutjanidae	0	0	0	0	**98**	**2**	0	0	0
*Acanthostracion quadricornis**	Ostraciidae	**95**	0	0	0	0	**5**	0	0	0
*Pomacanthus arcuatus**	Pomacanthidae	**95**	0	0	0	0	**5**	0	0	0
*Stegastes leucostictus**	Pomacentridae	**30**	0	0	0	0	**5**	0	**65**	0
Sparisoma chrysopterum	Scaridae	**55**	0	0	0	0	**30**	**5**	**10**	0
Pterois volitans	Scorpaenidae	0	0	0	0	**90**	0	0	0	**10**
Cephalopholis cruentata	Serranidae	0	0	0	0	**98**	**2**	0	0	0
Epinephelus guttatus	Serranidae	0	0	0	0	**98**	**2**	0	0	0
Epinephelus striatus	Serranidae	0	0	0	**30**	**70**	0	0	0	0
Mycteroperca bonaci	Serranidae	0	0	0	**19**	**79**	0	0	**2**	0
Sphyraena barracuda	Sphyraenidae	**3**	0	0	**57**	0	**10**	0	**30**	0
Sphoeroides testudineus	Tetraodontidae	**75**	0	**5**	0	0	**5**	**10**	**5**	0

[a]Phases shown as being produced by each fish species (except those marked with *; see below) were identified on basis of FTIR spectra as calcite, aragonite, monohydrocalcite, amorphous carbonate (ACMC), and brucite. Further classification (by $MgCO_3$ content) was based on compositional data (energy-dispersive X-ray spectroscopy; EDX).

*In the absence of phase data (i.e., samples were of insufficient size for FTIR or XRD analyses), phases produced by these species were inferred on the assumption that their precipitate morphotypes are of similar phase to comparable morphotypes found in samples in which phases are known. Confirmation of some of these phase inferences, and further phase classification (by $MgCO_3$ content) was based on EDX data.

[b]Volumetric abundance estimates are based on morphotype abundance estimates (Supplementary Table 2) and are converted from these on the basis of phases assigned to each morphotype (Supplementary Table 2) coupled with EDX compositional data. In a small number of cases, FTIR data (and XRD data – see Perry *et al.*[1]; Salter *et al.*[2]) indicate frequent occurrence of aragonite, but this phase is not seen through visual examinations (i.e., no aragonite morphotypes are seen). In these cases aragonite abundance is estimated on the basis of integrated peak areas of XRD patterns (following Davies and Hooper[3]).

Supplementary Table 2 The main precipitate morphotypes excreted by 22 Caribbean fish species, their estimated abundances per fish species, and the phases assigned to each based on results of FTIR and EDX spectroscopy.

	**Precipitate morphotype volumetric abundance (%)*[a,b]**									
**Fish species**	Monocrystalline ellipsoid HMC/VHMC (15–40 mol%)	Polycrystalline ellipsoid LMC	Rod/dumbbell (<5 µm)[c] HMC/VHMC (20–40 mol%)	Dumbbell (>5 µm) Arag/LMC/HMC (5–10 mol%)	Sphere (>5 µm) Arag/MHC/LMC HMC (5–20 mol%)	Needle/ spherulites Arag	Rhombohedron LMC	Plate/ rosette Brucite	Nanosphere (<1 µm) ACMC (20–37 mol%)	Amorphous ACMC (>70 mol%)
Albula vulpes	0	0	0	**4**	**60**	0	**1**	0	**20**	**15**
Platybelone argalus argalus	**40**	0	**25**	**12.5**	**7.5**	0	0	**5**	0	**10**
Bothus sp.	**100**	0	0	0	0	0	0	0	0	0
Eucinostomus sp.	0	0	0	**55**	**35**	0	0	0	0	**10**
Gerres cinereus	**70**	0	0	**15**	**15**	0	0	0	0	0
Haemulon flavolineatum	**100**	0	0	0	0	0	0	0	0	0
Haemulon sp.	0	0	**100**	0	0	0	0	0	0	0
Halichoeres bivittatus	0	0	0	**2.5**	**7.5**	0	**25**	0	0	**65**
Thalassoma bifasciatum	0	0	0	**2.5**	**7.5**	0	**25**	0	0	**65**
Lutjanus apodus	**98**	0	**2**	0	0	0	0	0	0	0
Ocyurus chrysurus	**100**	0	0	0	0	0	0	0	0	0
Acanthostracion quadricornis	0	0	0	**5**	**95**	0	0	0	0	0
Pomacanthus arcuatus	0	0	0	**80**	**20**	0	0	0	0	0
Stegastes leucostictus	0	0	0	**2.5**	**7.5**	0	**25**	0	0	**65**
Sparisoma chrysopterum	0	**35**	0	0	**35**	**10**	**10**	0	0	**10**
Pterois volitans	**90**	0	0	0	0	0	0	**10**	0	0
Cephalopholis cruentata	**100**	0	0	0	0	0	0	0	0	0
Epinephelus guttatus	**95**	0	0	0	0	0	**5**	0	0	0
Epinephelus striatus	**100**	0	0	0	0	0	0	0	0	0
Mycteroperca bonaci	**65**	0	**31**	0	0	0	**2**	0	0	**2**
Sphyraena barracuda	**5**	0	**62**	0	**1**	0	**2**	0	**20**	**10**
Sphoeroides testudineus	0	**15**	0	**10**	**65**	0	**5**	0	0	**5**

*See Salter *et al.*[2] for formal descriptions and example SEM images of each morphotype. Phases shown in grey are those assigned to each morphotype on the basis of FTIR spectroscopy and EDX spectroscopy.

[a]Volumetric abundance estimates are based on SEM observations and particle counts from both disaggregated and intact pellets.

[b]Where morphotypes are estimated to contribute <1 % of total volume, they are discarded from analyses (this is frequently the case for platy and rosette-forming brucite).

[c]Morphotypes are grouped together as part of a gradational sequence.

Supplementary Table 3 Habitat classification scheme for Bahamian shallow platform settings.

Habitat Type	Description	Example image
Sparse seagrass	Found in lagoonal environments where loose sediment substrate is sufficiently deep. Dominated by *Thalassia* but may contain *Syringodium* and *Halodule*. Small coral colonies may occasionally be present. Differentiated from other seagrass habitats by relatively low biomass (short plants, low density) and high amount of visible substrate (see Mumby *et al.*[4] for details).	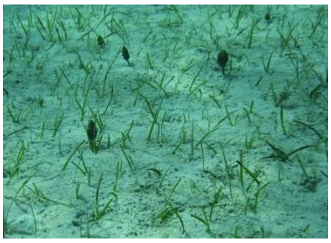
Medium density seagrass	As sparse seagrass, but with medium biomass (medium height plants, medium density) and medium amount of visible substrate (see Mumby *et al.*[4] for details).	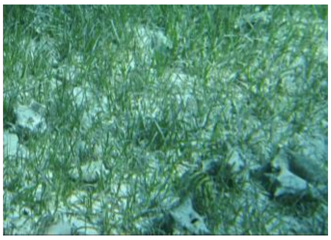
Dense seagrass	As sparse seagrass, but with high biomass (tall plants, high density) and limited amount of visible substrate (see Mumby *et al.*[4] for details).	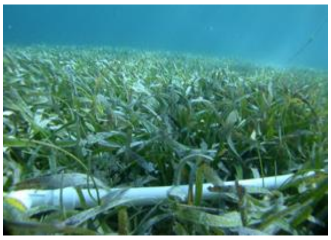
Batophora-dominated hard bottom	Found in low energy lagoonal environments. Abundant patches of *Batophora*, typically on a hard bottom with a limited covering of loose sediment. Other algae and some patches of seagrass are frequently present.	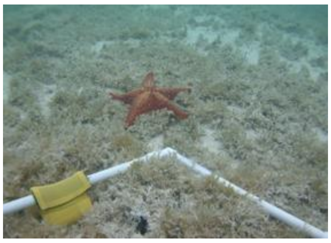
Sargassum on hard bottom	Found in medium energy lagoonal environments. Numerous *Sargassum* plants, typically on a hard bottom with a limited covering of loose sediment. In Andros the *Sargassum* plants reach 1 m tall. Other algae commonly present between *Sargassum* plants.	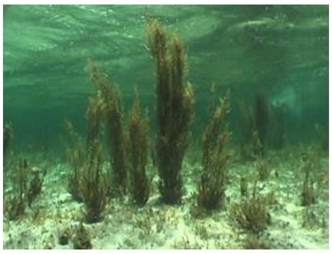
Macroalgal/ gorgonian plain on hard bottom	Found in both lagoonal and reefal environments, often covering large areas. Characterised by very low relief hard bottom with a mixed marcoalgal community. Algal coverage can be near to 100%. Alcyonacea (gorgonians) are typically either absent or sparse, although areas of dense cover are occasionally found.	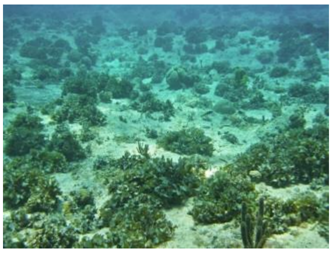
High-relief hard bottom	Similar benthic community to macroalgal/gorgonian plains, but with a higher relief hard bottom (typically large holes or undercut 'ledges'). Coral colonies often more frequent in this habitat type, which usually supports a diverse and abundant fish community.	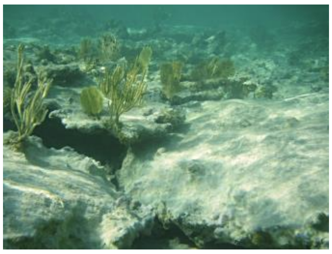

Supplementary Table 3 (continued) Habitat classification scheme for Bahamian shallow platform settings.

Habitat type	Description	Example image
Orbicella (formerly Montastraea) reef	Found in reefal environments at depths between approximately 5 and 15 m. *Orbicella annularis* is the most common coral species but there is a diverse benthic community including corals, sponges, gorgonians and algae. Typically supports a diverse and abundant fish community.	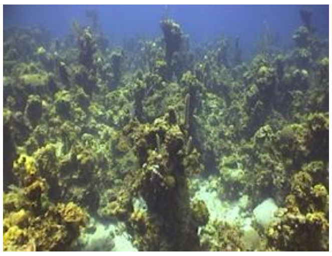
Acropora reef	Found in reef crest environments at depths between approximately 1 and 5 m. *Acropora* reef crests typically have high relief. *Acropora palmata* is the most common coral but there is a diverse benthic community including corals gorgonians and algae. Typically supports a diverse and abundant fish community.	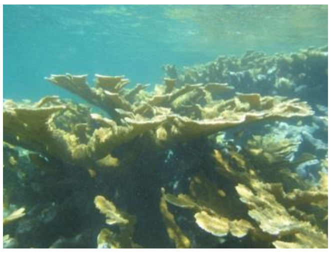
Patch reef	Found in lagoonal environments. Patch reefs are discrete hard bottom areas in the lagoon and can support a variety of benthic communities. Typically supports a diverse and abundant fish community.	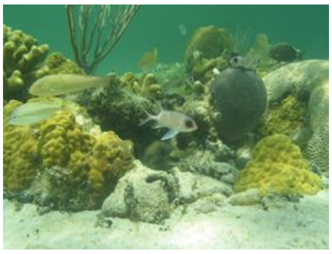
Spur-and-groove forereef	Forereefs that could be characterised as either of *Orbicella* or *Acropora* reefs, except that they are geomorphologically different – comprising a series of reef ridges separated by sand channels ranging from 1–5 m in width.	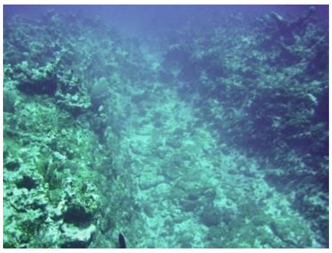
Fringing mangrove	Found on shorelines, tidal creeks and offshore islands. Survey sites were along the outer edge of stands of red mangrove (*Rhizophora mangle*). Prop roots are typically covered in a diverse community of sponges and algae. Mangrove stands usually support numerous juvenile and sub-adult fish, particularly species belonging to Haemulidae, Lutjanidae and Pomacentridae.	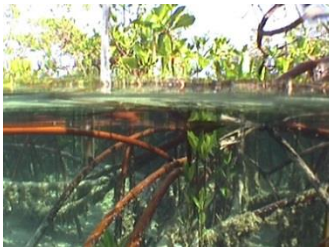
Bare sands	Found in both lagoonal and reefal environments. Characterised by clean sand, sometimes with a sparse algal community.	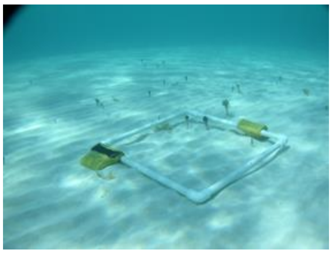

Supplementary Table 4 *Infrared spectroscopic data reported in literature for calcite, Mg-calcite, and aragonite (see Supplementary Table 3 footnote for details regarding methods, symbols, and references).*

Phase	Method	CO_3^{2-} vibrational modes						Other vibrations	Ref.
		ν_1 (cm^{-1})	ν_2 (cm^{-1})	ν_3 (cm^{-1})	ν_4 (cm^{-1})	$\nu_1 + \nu_3$ (cm^{-1})	$\nu_1 + \nu_4$ (cm^{-1})	Lattice modes	
Calcite, $CaCO_3$	ATR		871	NR	711				5
	ATR		868	1395	712				6
	ATR		872	1374	711	2511?	1795		7
	ATR		871	1392	711		1795		8
	KBr		877	1420	713			227, 311, 354	9
	KBr		877	NR	713				10
	KBr		876	NR	713				11
	KBr		870	1420	710				12
	KBr		870	1430	715				13
	KBr		874	1430	710				14
	KBr		876	1435	712	2545	1812		15
	KBr	1087?	881	1432	712				16
	KBr		877	1425	713				17
	KBr		877	1419	713				18
synthetic (syn)	KBr	1084	849§, 876	1426	712	2513	1798		19
natural (nat)	KBr	1083	849§, 876	1429	712	2513	1796		19
2 mol% $MgCO_3$ (syn)	KBr	1084	849§, 876	1435	713	2516	1798		19
2 mol% $MgCO_3$ (nat)	KBr		849§, 876	1431	713	2516	1797		19
6 mol% $MgCO_3$ (syn)	KBr	1086	849§, 874	1429	714.5	2519	1799		19
5 mol% $MgCO_3$ (nat)	KBr	1086	849§, 877	1435	713.3	2517	1798		19
11 mol% $MgCO_3$ (syn)	KBr	1086	849§, 875	1449	715.3	2525	1802		19
16 mol% $MgCO_3$ (nat)	KBr	1088	851§, 877	1429	719.5	2525	1802		19
23 mol% $MgCO_3$ (syn)	KBr	1084	876	1437	721.4	2529	1800		19
43 mol% $MgCO_3$ (nat)	KBr	1092	854§, 883	1451	731	2538	1815		19
Aragonite, $CaCO_3$	ATR	1084	856	1413	703, 714		1792		6
	KBr	1083	854	1440, 1488	700, 713			315, 273, 237, 220	9
	KBr	1083	859	NR	701, 713				10
	KBr	1080	855	1475	700, 715				13
	KBr	1080	855	1470	696, 710				14
	KBr	1085	875	1470	699, 712				15
	KBr	1087	866	1430, 1550	703, 715				16
	KBr	1085	858	1471	699, 713				17
	KBr	1083	857	1489, 1511	700, 713				18

Supplementary Table 5 *Reported infrared wavenumbers for hydrous Ca- and Mg-bearing carbonate (and related) phases (see Supplementary Table 3 footnote for details regarding methods, symbols, and references).*

Phase	Meth.	CO_3^{2-} vibrational modes					Other vibrations				Ref.
		ν_1 (cm^{-1})	ν_2 (cm^{-1})	ν_3 (cm^{-1})	ν_4 (cm^{-1})	$\nu_1 + \nu_4$ (cm^{-1})	HOH bend	OH^- stretch‡‡	OH^- stretch††	Lattice modes	
ACC, $(Ca,Mg)CO_3{\cdot}H_2O$	ATR	~1080	~865b	NR	711_{bh}	NR	~1645	~3400			5
*1:1 Mg:Ca solution	ATR	1072	863	1396, ~1470			1645	~3300			6
*2:1 Mg:Ca solution	ATR	1073	856	1385, ~1450			1642	~3300			6
*4:1 Mg:Ca solution	ATR	1074	861	1382, ~1450			1645	~3300			6
*10:1 Mg:Ca solution	ATR	1073	858	1375, ~1450			1645	~3300			6
	KBr	1067	864b	1425, 1490	725_{bh}	1793	1640	3230, 3375			9
	KBr	NR	876	NR	713_{bh}			~3500			11
	KBr	1075	860	1405, 1480	705, 735_{bh}		1645				12
	KBr	NR**	866	1406, 1474			1655	NR			20
	KBr	NR**	866	1420, 1474			1653	NR			21
	KBr	NR**	873	1425, 1470			1635	~3430			21
Ikaite, $CaCO_3{\cdot}6H_2O$	$NaCl^{\dagger}$	1085	876	1411, 1425	720, 743		1644	3216, 3404, 3468		800?	22
Monohydrocalcite	$NaCl^{\dagger}$	1063	872	1401, 1492	698, 762			3236, 3327, 3400			22
$CaCO_3{\cdot}H_2O$	NR	1068	873	1408, 1487	700, 727, 766		1700	3235			23
Nesquehonite	$NaCl^{\dagger}$	1099	854	1427, 1467, 1518	719			3350, 3460, 3508			22
$MgCO_3{\cdot}3H_2O$	KBr	1097	~855	1415, 1470, 1518	~710		1640	~3400	3568		24
$Mg(OH)(HCO_3){\cdot}2H_2O^{\ddagger}$	ATR	1098	855	1419, 1439, 1462	710		1636, 1683	3012–3483*	3560, 3605		25
Dypingite	ATR	1071	852, 882	1380–1508*	755, 799			~3400	3647, 3686	948, 1008	26
$Mg_5(CO_3)_4(OH)_2{\cdot}5H_2O$	ATR	1078	NR	1312–1585*	NR			~3400	3649, 3682	947, 1012	26
Artinite	ATR	1076	844	1376–1535*	NR			~3400	3649, 3685	947, 1017	26
$Mg_2CO_3{\cdot}(OH)_2{\cdot}3H_2O$	ATR	1083	NR	1325–1439*	722, 762			~3400	3651, 3685	952, 995	26
Hydromagnesite	NR	1119	800, 854, 885	1424, 1482	601		1644	3448	3648		27
$Mg_5(CO_3)_4{\cdot}(OH)_2{\cdot}4H_2O$	KBr	1110, 1120	~800, ~860, ~900	1420, 1475, 1515	~600		~1650	~3100, 3463, 3525	3658		24
Brucite	KBr								3700–3704		28
$Mg(OH)_2$	NR								3698	440, 565, 627	29
	KBr								3698	445, 571, 645	30

Supplementary Table 6 *Reported infrared wavenumbers for selected additional anhydrous Ca- and Mg-bearing carbonate phases (see footnote for details regarding methods, symbols).*

Phase	Method	CO_3^{2-} vibrational modes						Other vibrations	Ref.
		ν_1 (cm^{-1})	ν_2 (cm^{-1})	ν_3 (cm^{-1})	ν_4 (cm^{-1})	$\nu_1 + \nu_3$ (cm^{-1})	$\nu_1 + \nu_4$ (cm^{-1})	Lattice modes	
Vaterite, $CaCO_3$	ATR	1085	868	NR	747				6
	KBr	1089	873, 877	1418, 1487	738, 746			383, 332, 282, 250, 232	9
	KBr	1085	870	1420, 1490	750				13
	KBr	1090	878	1450	741				16
	KBr	1089	877	1450	744				18
Dolomite, $CaMg(CO_3)_2$	KBr		881	1439	729				17
	KBr		881	1450	730	2530	1818		15
	KBr		881	1437	728	2524	1823		31
synthetic	KBr	1100	853[§], 883	1445	729	2529	1818		19
natural	KBr	1101	853[§], 882	1443	728	2526	1821		19
Huntite, $Mg_3Ca(CO_3)_4$	KBr	1112	870, 893	1445, 1511	743				17
	KBr	1113	869, 891	1452, 1467, 1512, 1530	743	2544, 2583	1828		19
Magnesite, $MgCO_3$	KBr		887	1450	748		1818		15
synthetic	KBr	1108	855[§], 886	1448	748	2537	1831		19
natural	KBr	1113	855[§], 885	1456	748	2535	1827		19

Cell values Empty cell = peak not present; NR = peak position not reported. **Method** KBr = Transmission FTIR, sampled mounted in KBr disc; ATR = Attenuated internal reflectance FTIR; NaCl[†] = Transmission FTIR, samples mounted in NaCl windows and analysed at -80 °C. **OH^- stretch** [††]OH^- strongly bonded in hydroxy mineral phases, generally very sharp peaks; [‡‡]OH^- associated with water in various states, generally broad peaks. **Additional notes** *numerous peaks within this range; [‡]a different formula is proposed for nesquehonite based this on IR spectrum. The authors observe additional peaks (not shown in the table) at 934 cm^{-1}, which they assign to the MgOH deformation mode, and in the region 624–680 cm^{-1}, 836 cm^{-1}, 1027 and 1052 cm^{-1}, and 1511, 1528, and 1584 cm^{-1}, these being attributed to ν_4, ν_2, ν_3, and ν_1 vibrational modes of HCO_3^-, respectively; **If present, this peak is obscured by a relatively intense phosphate peak; [§]Weak satellite peak assigned to vibration of $^{13}CO_3^{2-}$; $_{bh}$ = peak present as an indistinct broad hump.

Supplementary Note 1: *Further discussion regarding the fates of brucite and ACMC.*

Solubility data based on analyses involving a wide range of biogenically- and synthetically-derived materials are widely available for low-Mg calcite, high-Mg calcite, and aragonite; these generally being in good agreement[32], with the exception of data for high Mg-calcite (see Morse and Mackenzie[32] for a discussion of the problems associated with determining the solubility of this phase). These data, along with available solubility data for monohydrocalcite, were used to inform the discussion in the main text about the possible fates of equivalent fish-derived phases. Solubility data for amorphous carbonates and brucite, however, are less widely available, and a variety of factors may influence the post-excretion fates of these phases.

Brucite is scarce in marine settings; its occurrence being documented mainly from active portions of deep-sea hydrothermal vent structures[33–35] and in shallow-water settings in association with skeletal carbonates of living specimens of some corals and red algae[36–38]. In each of these examples, precipitation of brucite is thought to have been driven by localised high pH and high Mg^{2+}/Ca^{2+} ratios, and its precipitation in the piscine gut, where elevated pH levels (up to 9.2) and high concentrations of Mg^{2+} (up to 200 mM) are reported[39], is thus readily explained. However, we note that brucite is extremely rare in marine sediments and is reportedly absent both from submerged Holocene corals equivalent to living specimens containing brucite[36], and from extinct portions of hydrothermal vent systems[34]. These observations suggest that brucite is not preserved for long durations in open marine conditions; an assertion supported by thermodynamic calculations which suggest seawater is undersaturated with respect to brucite[35]. In addition, solubility experiments involving skeletal parts of the red algae, *Goniolithon* and *Neogoniolithon* (comprising ~18 mol% Mg-calcite and subsidiary brucite), reportedly yield initially rapid dissolution in aqueous solution "…*of an exceedingly soluble phase (presumably brucite)*…" prior to a lowering of dissolution rate, presumably following the complete removal of brucite[40–42]. These observations suggest brucite is thermodynamically unstable in seawater and is considerably more soluble than 18 mol% Mg-calcite, and it is thus likely that, post-excretion, brucite associated with fish-derived carbonates will be rapidly removed via dissolution.

Solubility data for hydrated amorphous calcium carbonate (ACC) indicate that it is a highly unstable phase, with reported solubility products ranging from 4.0×10^{-7} to 9.1×10^{-7} at 25 °C[43,44] making it ~120–270 times more soluble than calcite. However, application of these data to fish-derived amorphous carbonates (ACMC) is complicated by the fact that they typically contain magnesium (20–37 mol% $MgCO_3$ in nanospheres and >70 mol% $MgCO_3$ in material that lacks definable form) and, in some cases, phosphate[45,46], both of which are known to stabilise amorphous carbonates to some extent[20]. Nevertheless, it is widely held that amorphous carbonates, regardless of composition, are highly unstable and will either dissolve or crystallise within minutes to days of removal from stable conditions[20,47,48], and we therefore assume that, post-excretion, fish-derived ACMC will follow one or both of these pathways. The results of seawater exposure experiments help to resolve this issue: fish-derived ACMC (similar to that described herein) left in seawater for 1 hr reportedly dissolves very rapidly[45]. In similar experiments we have noted rapid increases in solution pH, also suggesting dissolution has occurred. However, the phases and compositions of solid matter that remains at the end of such experiments have yet to be documented, and it therefore remains to be determined whether fish-derived ACMC undergoes wholescale dissolution upon exposure to seawater, or partial dissolution and partial crystallisation.

Supplementary Methods 1: *Production–biomass calibration model parameters.*

Quadratic term removed from model.

Linear mixed-effects model fit by REML
Data: NULL

AIC	BIC	logLik
443.1258	450.7739	-217.5629

Random effects:
Formula: ~1 | Family

	(Intercept)	Residual
StdDev:	14.92101	14.53954

Fixed effects: Production ~ Log biomass

	Value	Std.Error	DF	t-value	p-value
(Intercept)	62.03252	9.109466	38	6.809677	0.0000
Log biomass	-5.95921	1.830744	38	-3.255077	0.0024

Family specific intercepts:

Barracuda (Sphyraenidae)	51.92117
Bonefish (Albulidae)	56.63013
Damsel (Pomacentridae)	37.26386
Grouper (Serranidae)	61.86581
Grunt (Haemulidae)	84.77622
Lionfish (Scorpaenidae)	52.45873
Mojarra (Gerreidae)	63.46057
Mullet (Mugilidae)	62.42459
Needlefish (Belonidae)	80.13719
Other	65.14246
Puffer (Tetraodontidae)	52.01106
Snapper (Lutjanidae)	67.90761
Wrasse (Labridae)	70.42340

Supplementary References

1. Perry, C. T. *et al*. Fish as major carbonate mud producers and missing components of the tropical carbonate factory. *Proc. Natl. Acad. Sci. USA* **108,** 3865–3869 (2011).
2. Salter, M. A., Perry, C. T. & Wilson R. W. Production of mud-grade carbonates by marine fish: crystalline products and their sedimentary significance. *Sedimentology* **59,** 2172–2198 (2012).
3. Davies, T. T. & Hooper, P. R. The determination of the calcite:aragonite ratio in mollusc shells by X-ray diffraction. *Mineral. Mag.* **33,** 608–612 (1962).
4. Mumby, P. J. *et al*. A visual assessment technique for estimating seagrass standing crop. *Aquatic Conserv: Mar. Freshw. Ecosyst.* **7,** 239–251 (1997).
5. Gong, Y. U. T. *et al.* Phase transitions in biogenic amorphous calcium carbonate. *Proc. Natl. Acad. Sci. USA* **109,** 6088–6093 (2012).
6. Loste, E., Wilson, R. M., Seshadri, R. & Meldrum, F. C. The role of magnesium in stabilising amorphous calcium carbonate and controlling calcite morphologies. *J. Cryst. Growth* **254,** 206–218 (2003).
7. Bullen, H. A., Oehrle, S. A., Bennett, A. F., Taylor, N. M. & Barton, H. A. Use of attenuated total reflectance Fourier transform infrared spectroscopy to identify microbial metabolic products on carbonate mineral surfaces. *Appl. Environ. Microbiol.* **74,** 4553–4559 (2008).
8. Boels, L., Wagterveld, R. M., Mayer, M. J. & Witkamp, G. J. Seeded calcite sonocrystallization. *J. Cryst. Growth* **312,** 961–966 (2010).
9. Andersen, F. A. & Brečević, L. Infrared spectra of amorphous and crystalline calcium carbonate. *Acta Chem. Scand.* **45,** 1018–1024 (1991).
10. Compere, E. L., Jr. & Bates, J. M. Determination of calcite:aragonite ratios in mollusc shells by infrared spectra. *Limnol. Oceanogr.* **18,** 326–331 (1973).
11. Politi, Y., Arad, T., Klein, E., Weiner, S. & Addadi, L. Sea urchin spine calcite forms via a transient amorphous calcium carbonate phase. *Science* **306,** 1161–1164 (2004).
12. Dupuis, T., Ducloux, J., Butel, P. & Nahon, D. Etude par spectrographie infrarouge d'un encroutement calcaires sous Galet. Mise en evidence et modelisation experimentale d'une suite minerale evolutive a partir de carbonate de calcium amorphe. *Clay Miner.* **19,** 605–614 (1984).
13. Sato, M. & Matsuda, S. Structure of vaterite and infrared spectra. *Z. Kristallogr.* **129,** 405–410 (1969).
14. Nakamoto, K., Fujita, J., Tanaka, S. & Kobayashi, M. Infrared spectra of metallic complexes. IV. Comparison of the infrared spectra of unidentate and bidentate metallic complexes. *J. Am. Chem. Soc.* **79,** 4904–4908 (1957).
15. Huang C. K. & Kerr P. F. Infrared study of the carbonate minerals. *Am. Mineral.* **43,** 311–324 (1960).
16. Weir, C. E. & Lippincott, E. R. Infrared studies of aragonite, calcite, and vaterite type structures in the borates, carbonates, and nitrates. *J. Res. NBS – A: Phys. Chem.* **65A,** 173–183 (1961).
17. Adler, H. H. & Kerr, P. F. (1963) Infrared spectra, symmetry and structure relations of some carbonate minerals. *Am. Mineral.* **48,** 839–853 (1963).
18. Sterzel, W. Der Einfluß der kristallstruktur auf die kopplung der v_2-schwingung von carbonationen. *Z. Anorg. Allg. Chem.* **368,** 308–316 (1969).
19. Böttcher, M. E., Gehlken, P. -L. & Steele, D. F. Characterization of inorganic and biogenic magnesian calcites by Fourier Transform infrared spectroscopy. *Solid State Ionics* **101–103,** 1379–1385 (1997).

20. Raz, S., Testeniere, O., Hecker, A.,Weiner, S. & Luquet, G. Stable amorphous calcium carbonate is the main component of the calcium storage structures of the crustacean *Orchestia cavimana*. *Biol. Bull.* **203,** 269–274 (2002).
21. Aizenberg, J., Lambert, L., Addadi, L. & Weiner, S. Stabilization of amorphous calcium carbonate by specialized macromolecules in biological and synthetic precipitates. *Adv. Mater.* **8,** 222–226 (1996).
22. Coleyshaw, E. E., Crump, G. & Griffith, W. P. Vibrational spectra of the hydrated carbonate minerals ikaite, monohydrocalcite, lansfordite and nesquehonite. *Spectrochim. Acta A* **59,** 2231–2239 (2003).
23. Neumann, M. & Epple, M. Monohydrocalcite and its relationship to hydrated amorphous calcium carbonate in biominerals. *Eur. J. Inorg. Chem.* **2007,** 1953–1957 (2007).
24. White, W. B. Infrared characterization of water and hydroxyl ion in the basic magnesium carbonate minerals. *Am. Mineral.* **56,** 46–53 (1971).
25. Frost, R. L. & Palmer, S. J. Infrared and infrared emission spectroscopy of nesquehonite $Mg(OH)(HCO_3)\cdot 2H_2O$ – implications for the formula of nesquehonite. *Spectrochim. Acta A* **78,** 1255–1260 (2011).
26. Frost, R. L., Bahfenne, S., Graham, J., & Reddy, B. J. The structure of selected magnesium carbonate minerals – a near infrared and mid-infrared spectroscopic study. *Polyhedron* **27,** 2069–2076 (2008).
27. Janet, C. M., Viswanathan, B., Viswanath, R. P. & Varadarajan, T. K. Characterization and photoluminescence properties of MgO microtubes synthesized from hydromagnesite flowers. *J. Phys. Chem. C* **111,** 10267–10272 (2007).
28. Ahlrichs, J. L. Hydroxyl stretching frequencies of synthetic Ni-, Al-, and Mg-hydroxy interlayers in expanding clays. *Clays Clay Miner.* **16,** 63–71 (1968).
29. Frost, R. L. & Kloprogge, J. T. Infrared emission spectroscopic study of brucite. *Spectrochim. Acta A* **55,** 2195–2205 (1999).
30. Schroeder, P. A. Infrared spectroscopy in clay science. In: *Teaching in Clay Science,* CMS Workshop Lectures, 11 (Eds. A. Rule and S. Guggenheim). The Clay Mineral Society, Aurora, CO, 181–206 (2002).
31. Ramasamy, V., Ponnusamy, V., Sabari, S., Anishia, S. R. & Gomathi, S. S. Effect of grinding on the crystal structure of recently excavated dolomite. *Indian J. Pure Appl. Phys.* **47,** 586–591 (2009).
32. Morse, J. W. & Mackenzie, F. T. *Geochemistry of Sedimentary Carbonates*. (Elsevier, Amsterdam, 1990).
33. Kelley, D.S. *et al.* An off-axis hydrothermal vent field discovered near the Mid-Atlantic Ridge at 30° N. *Nature* **412,** 150–157 (2001).
34. Kelley, D. S. *et al*. A serpentinite-hosted ecosystem: the Lost City hydrothermal field. *Science* **307,** 1428–1434 (2005).
35. Früh-Green G. L. *et al*. 30,000 years of hydrothermal activity at the Lost City vent field. *Science* **301,** 495–498 (2003).
36. Nothdurft, L. D. *et al*. Brucite microbialites in living coral skeletons: Indicators of extreme microenvironments in shallow-marine settings. *Geology* **33,** 169–172 (2005).
37. Buster, N. A. & Holmes, C. W. Magnesium content within the skeletal architecture of the coral *Montastraea faveolata*: locations of brucite precipitation ans implications for fine-scale data fluctuations. *Coral Reefs* **25,** 243–253 (2006).
38. Schmalz, R. F. Kinetics and diagenesis of carbonate sediments. *J. Sed. Res.* **37,** 60–67 (1967).

39. Wilson, R. W., Wilson, J. M. & Grosell, M. Intestinal base secretion by marine teleost fish – why and how? *Biochim. Biophys. Acta* **1566,** 182–193 (2002).
40. Walter, L. M. & Hanor, J. S. Orthophosphate: effect on the relative stability of aragonite and magnesian calcite during early diagenesis. *J. Sed. Pet.* **49,** 0937–0944 (1979).
41. Walter, L. M. & Morse, J. W. Magnesian calcite stabilities: a re-evaluation. *Geochim. Cosmochim. Acta* **48,** 1059–1069 (1984).
42. Walter, L. M. & Morse, J. W. The dissolution kinetics of shallow marine carbonates in seawater: a laboratory study. *Geochim. Cosmochim. Acta* **49,** 1503–1513 (1985).
43. Brečević, L. & Nielsen, A. E. Solubility of amorphous calcium carbonate. *J. Cryst. Growth* **98,** 504–510 (1989).
44. Clarkson, J. R., Price, T. J. & Adams, C. J. Role of metastable phases in the spontaneous precipitation of calcium carbonate. *J. Chem. Soc. Faraday Trans.* **88,** 243–249 (1992).
45. Foran, E., Weiner, S. & Fine, M. Biogenic fish-gut calcium carbonate is a stable amorphous phase in the gilt-head seabream, *Sparus aurata*. *Sci. Rep.* **3,** article no. 1700 (2013).
46. Salter, M. A. *The Production and Preservation of Fish-derived Carbonates in Shallow Tropical Marine Carbonate Provinces.* Unpublished Ph.D thesis, Manchester Metropolitan University (2013).
47. Beniash, E., Aizenberg, J., Addadi, L. & Weiner, S. Amorphous calcium carbonate transform into calcite during sea urchin larval spicule growth. *Proc. Roy. Soc. Lond.* **264B,** 461–465 (1997).
48. Radha, A. V., Forbes, T. Z., Killian, C. E., Gilbert, P. U. P. A. & Navrotsky, A. Transformation and crystallization energetics of synthetic and biogenic amorphous calcium carbonate. *Proc. Natl. Acad. Sci. USA* **107,** 16438–16443 (2010).
